# RNA-Seq Analysis Identifies New Genes Regulated by the Histone-Like Nucleoid Structuring Protein (H-NS) Affecting *Vibrio cholerae* Virulence, Stress Response and Chemotaxis

**DOI:** 10.1371/journal.pone.0118295

**Published:** 2015-02-13

**Authors:** Hongxia Wang, Julio C. Ayala, Jorge A. Benitez, Anisia J. Silva

**Affiliations:** 1 Morehouse School of Medicine Department of Microbiology, Biochemistry and Immunology, Atlanta, Georgia, United States of America; 2 University of Alabama at Birmingham Department of Microbiology, Birmingham, Alabama, United States of America; 3 State Key Laboratory for Infectious Disease Prevention and Control, and National Institute for Communicable Disease Control and Prevention, Chinese Center for Disease Control and Prevention, Changping, Beijing, China

## Abstract

The histone-like nucleoid structuring protein (H-NS) functions as a transcriptional silencer by binding to AT-rich sequences at bacterial promoters. However, H-NS repression can be counteracted by other transcription factors in response to environmental changes. The identification of potential toxic factors, the expression of which is prevented by H-NS could facilitate the discovery of new regulatory proteins that may contribute to the emergence of new pathogenic variants by anti-silencing. *Vibrio cholerae hns* mutants of the El Tor biotype exhibit altered virulence, motility and environmental stress response phenotypes compared to wild type. We used an RNA-seq analysis approach to determine the basis of the above *hns* phenotypes and identify new targets of H-NS transcriptional silencing. H-NS affected the expression of 18% of all predicted genes in a growth phase-dependent manner. Loss of H-NS resulted in diminished expression of numerous genes encoding methyl-accepting chemotaxis proteins as well as chemotaxis toward the attractants glycine and serine. Deletion of *hns* also induced an endogenous envelope stress response resulting in elevated expression of *rpoE* encoding the extracytoplamic sigma factor E (σ^E^). The RNA-seq analysis identified new genes directly repressed by H-NS that can affect virulence and biofilm development in the El Tor biotype cholera bacterium. We show that H-NS and the quorum sensing regulator HapR silence the transcription of the *vieSAB* three-component regulatory system in El Tor biotype *V. cholerae*. We also demonstrate that H-NS directly represses the transcription of *hlyA* (hemolysin), *rtxCA* (the repeat in toxin or RTX), *rtxBDE* (RTX transport) and the biosynthesis of indole. Of these genes, H-NS occupancy at the *hlyA* promoter was diminished by overexpression of the transcription activator HlyU. We discuss the role of H-NS transcriptional silencing in phenotypic differences exhibited by *V. cholerae* biotypes.

## Introduction

Cholera is an acute water-borne diarrheal disease caused by *Vibrio cholerae* of serogroups O1 and O139. *V*. *cholerae* O1 can be divided in two biotypes, classical and El Tor [1], which differ in the expression and regulation of major virulence factors [1–3]. One major difference is that El Tor biotype strains require special conditions for the *in vitro* expression of cholera toxin (CT) and the toxin co-regulated pilus (TCP), required for intestinal colonization [4]. Other regulators and virulence factors that differ in their expression between biotypes are the VieSAB three-component regulatory system, [3], hemolysin [1] and the repeat in toxin (RTX) [2].

The cholera bacterium has evolved to sense and effectively colonize disparate ecological niches, the aquatic environment and the human small intestine. In the aquatic environment, *Vibrios* are subject to numerous physical, chemical and biological stresses which include nutrient limitation, extreme temperatures, oxidative stress, bacteriophage infection and protozoan grazing [5–8]. During infection, *Vibrios* are subject to a myriad of additional host-induced stresses such as low pH, bile and the activity of antimicrobial peptides. Transitions between the aquatic environment and the human small intestine require the regulation, integration and fine tuning of numerous cellular processes, such as virulence gene expression, motility and biofilm development. The formation of biofilm communities is critical for *V*. *cholerae* survival and persistence in nature as well as in disease transmission [5, 9–11].

The histone-like nucleoid structuring protein (H-NS) is a nucleoid associated protein and transcriptional repressor [12]. H-NS belongs to a family of small nucleoid associated proteins that include the factor for inversion stimulation (FIS), and the integration host factor (IHF) [13]. H-NS consists of an N-terminal oligomerization domain connected by a flexible linker to a nucleic acid binding domain [12–15]. Both DNA binding and oligomerization are required for the biological activities of H-NS, which include DNA condensation and the regulation of transcription [16, 17]. In transcription regulation, H-NS negatively affects gene expression by binding to promoters exhibiting AT-rich, highly curved DNA regions [18–20]. Repression by H-NS can be relieved in response to environmental cues that activate the expression of other regulators whose binding site overlaps that of H-NS [13, 21]. For example, transcriptional silencing of *V*. *cholerae tcpA* and *ctxA* promoters by H-NS is antagonized by the AraC-like transcriptional regulator ToxT and the IHF [22–24]. Also, H-NS repression at the *flrA* and *rpoN* promoters in late stationary phase cells is counteracted by RpoS and IHF [25].

Mutations that inactivate *hns* are highly pleiotropic [26, 27]; the mutants form small colonies in agar plates; exhibit diminished growth rate, and altered cell morphology. The El Tor biotype *hns* mutants express CT and TCP under ToxR non-permissive conditions (LB, 30°C) [27, 28]. Although El Tor biotype *hns* mutants are flagellated, they show reduced motility in swarm agar plates [25]. In addition, we have shown that *hns* mutants exhibit enhanced resistance to low pH and hydrogen peroxide [27]. To determine the basis of the *hns* mutant altered virulence gene expression, its reduced swarm halo in soft agar plates, and altered stress responses [25, 27], we conducted an RNA-seq analysis of an El Tor biotype *hns* mutant in exponential and stationary phases. Here we show that H-NS affects the expression of 18% of the *V*. *cholerae* genome in a growth phase-dependent manner. Loss of H-NS resulted in diminished chemotaxis and induced an endogenous envelope/oxidative stress. Further, we demonstrate that H-NS directly represses the expression of (i) the *vieSAB* operon, hemolysin, and the RTX toxin, and (ii) indole biosynthesis, an activator of biofilm development.

## Materials and Methods

### Strains, plasmids, primers and culture media

The strains, plasmids, and oligonucleotide primers used in this study are listed and briefly described in Tables 1 and 2. *V*. *cholerae* and *Escherichia coli* strains were grown in LB medium at 37°C with agitation (225 rpm). *E*. *coli* strain TOP10 (Invitrogen) and S17–1λpir [29] were used for plasmid propagation and cloning. To measure the expression of CT genes, *V*. *cholerae* strains were grown in LB medium or AKI as described in [30]. To measure chemotaxis, *V*. *cholerae* strains were grown in TG medium (1% tryptone, 0.5% NaCl and 0.5% glycerol) at 30°C. Culture media were supplemented with ampicillin (Amp, 100 μg/mL), kanamycin (Km, 25 μg/mL), chloramphenicol (Cm, 2.5 μg/mL), streptomycin (Str, 100 μg/mL), 2,2′-dipyridyl (0.2 mM), polymyxin B (PolB, 100 units/mL), isopropyl-β-D-thiogalactopyranoside (IPTG, 20 μg/mL), L-arabinose (0.02% w/v) or 5-bromo-4-chloro-3-indolyl-β-D-galactopyranoside (X-Gal, 20 μg/mL) as required and described in the figure legends.

**Table 1:** Strains and Plasmid.

Strain or plasmid	Description	Source or reference
**Strains**		
C7258	Wild type O1; El Tor biotype	Peru, 1991
C7258Δ*lacZ*	C7258 *lacZ* deletion mutant	[27]
AJB51Δ*lacZ*	C7258Δ*lacZ* Δ*hapR*	[27]
O395Δ*lacZ*	Classical biotype O395 *lacZ* deletion mutant	[35]
AJB608	O395Δ*lacZ* Δ*hns*::km	This study
AJB80	C7258 Δ*lacZ* Δ*hns*::km	[27]
AJB700	C7258 Δ*lacZ* Δ*hapR* Δ*hns*::km	This study
C7258HNS-FLAG	C7258 *hns*::*hns*-FLAG	[25]
AJB606	C7258 Δ*lacZ*Δ*vieA*	This study
AJB607	C7258 Δ*lacZ* Δ*hns*::km Δ*vieA*	This study
AJB600	C7258 Δ*lacZ* Δ*vieSAB*	This study
AJB601	C7258 Δ*lacZ* Δ*hns*::km Δ*vieSAB*	This study
AJB602	C7258 Δ*lacZ* Δ*hlyU*::cm	This study
AJB603	C7258 Δ*lacZ* Δ*hns*::km Δ*hlyU*::cm	This study
WL7258ΔlacZ	C7258 Δ*lacZ* Δ*crp*	[27]
AJB604	C7258 Δ*lacZ* Δ*crp* Δ*hns*::Km	This study
**Plasmids**		
pCR2.1	Bacterial expression vector for TA cloning (Amp^R^)	Invitrogen
pBAD33	pACYC184 *ori araC* p_araBAD_, Cm^R^	[45]
pBAD-HlyU-HIS	pBAD33 containing the *hlyU* ORF 3’ expressed from the arabinose promoter	This study
pCAT	Chloramphenicol acetyl transferase (*cat*) gene in pUC219	B. Kan
pUC-VieA-UP	0.56 kb *Xba*I-*Sal*I DNA fragment 5’ of *vieA* in pUC19	This study
pUC-VieA-DN	0.57 kb *Sal*I-*Sph*I DNA fragment 3’ of *vieA* in pUC19	This study
pUCΔVieA	*Xba*I-*Sal*I and SalI-SphI fragments flanking *vieA* in pUC19	
pCVDΔVieA	*Xba*I-*Sph*I fragment harboring *vieA* deletion in pCVD442	
pUC-VieSAB-UP	0.52 kb *Xba*I-*Sal*I DNA fragment 5’ of *vieSAB* in pUC19	This study
pUC-VieSAB-DN	0.47 kb *Sal*I-*Sph*I DNA fragment 3’ of *vieSAB* in pUC19	This study
pUCΔVieSAB	*Xba*I-*Sal*I and SalI-SphI fragments flanking *vieSAB* in pUC19	This study
pCVDΔVieSAB	*Xba*I-*Sph*I fragment harboring *vieSAB* deletion in pCVD442	This study
pUC-HlyU-UP	0.52 kb *Sac*I-*BamH*I DNA fragment 5’ of *hlyU* in pUC19	This study
pUC-HlyU-DN	0.48 kb *BamH*I-*Sph*I DNA fragment 3’ of *hlyU* in pUC19	This study
pUCΔHlyU	*Sac*I-*BamH*I and *BamH*I-*Sph*I fragments flanking *hlyU* in pUC19	This study
pUCΔHlyU- Cm	0.8 kb *BamH*I fragment encoding *cat* gene in pUCΔHlyU	This study
pCVDΔHlyU- Cm	*Sac*I-*Sph*I fragment harboring *vieSAB* deletion and *cat* insertion in pCVD442	This study
pCVDΔHNSK	DNA fragment carrying *hns* deletion and km insertion in pCVD442	[27]
pTT3	*Xba*I-*Pst*I fragment encoding *rrnBT*_*1*_ *T*_*2*_ transcription terminator in pUC19	[38]
pTT3VieSAB^ET^	633 bp *Sph*I-*Hind*III El Tot biotype *vieSAB* promoter fragment in pTT3	This study
pTT3VieSAB^CL^	633 bp *Sph*I-*Hind*III classical biotype *vieSAB* promoter fragment in pTT3	This study
pTT3HlyA	540 bp *Sph*I-*Hind*III *hlyA* promoter fragment in pTT3	This study
pTT3RtxCA	250 bp *Sph*I-*Hind*III *rtxCA* promoter fragment in pTT3	This study
pTT3RtxBDE	230 bp *Sph*I-*Hind*III *rtxBDE* promoter fragment in pTT3	This study
pTT3RpoE^P1^	190 bp *Sph*I-*Hind*III *rpoE* P1 promoter fragment in pTT3	This study
pTT3RpoE^P2^	180bp *Sph*I-*Hind*III *rpoE* P2 promoter fragment in pTT3	This study
pKRZ1	Plasmid source for promoterless lacZ gene	[39]
pVieSAB^ET^-LacZ	1040 bp *Kpn*I-*Hind*III *rrnBT*_*1*_ *T*_*2*_-*vieSAB* ^ET^ cassette ligated to promoterless *lacZ* gene in pKRZ1	This study
pVieSAB^CL^-LacZ	1040 bp *Kpn*I-*Hind*III *rrnBT*_*1*_ *T*_*2*_-*vieSAB* ^CL^ cassette ligated to promoterless *lacZ* gene in pKRZ1	This study
pHlyA-LacZ	1001 bp *Kpn*I-*Hind*III *rrnBT*_*1*_ *T*_*2*_-*hlyA* cassette fused to *lacZ* gene	This study
pRtxCA-LacZ	704 bp *Kpn*I-*Hind*III fragment *rrnBT*_*1*_ *T*_*2*_-*rtxCA* cassette fused to lacZ gene	This study
pRtxBDE-LacZ	684 bp *Kpn*I-*Hind*III *rrnBT*_*1*_ *T*_*2*_-*rtxBDE* cassette fused to *lacZ* gene	This study
pRpoE^P1^-LacZ	641 bp *Kpn*I-*Hind*III *rrnBT*_*1*_ *T*_*2*_-*rpoE* ^P1^ cassette ligated *lacZ* gene	This study
pRpoE^P2^-LacZ	625 bp *Kpn*I-*Hind*III *rrnBT*_*1*_ *T*_*2*_-*rpoE* ^P2^ cassette ligated to *lacZ* gene	This study
pTT5	*Kpn*I-*BamH*I *rrnBT*_*1*_ *T*_*2*_ transcription terminator fragment in pUC19	This study
pTT5TnaA	1140 bp *BamH*I-*Xba*I *tnaA* promoter fragment in pTT5	This study
pTnaA-LacZ	1570 bp *Kpn*I-*Xba*I *rrnBT*_*1*_ *T*_*2*_-*tnaA* cassette ligated to *lacZ* gene	This study
pCTX-LacZ	*rrnBT*_*1*_ *T*_*2*_-*ctxA* promoter fragment ligated to *lacZ* gene	[35]
pRpoE-LacZ	*rrnBT*_*1*_ *T*_*2*_ and *rpoE* P1P2 promoter in tandem fused to *lacZ* gene	[35]

**Table 2:** Primers.

Primer	Sequence (5’→3’)
70-F	GAAGCATGCCCGCATCGATTGAGCAGAC
70-R	GCGAAGCTTTTTATATAGTGGTGAATGC
70-P1-F1	CCGCATCGATTGAGCAGACATG
70-P1-R1	TTTATATAGTGGTGAATGCAGCG
357F	CCTACGGGAGGCAGCAG
926R	CCGTCAATTCMTTTRAGT
1448-F1	GCAACCATCAGACAAATTGCAC
1448-F2	GTATTCCCTCAATTTCACACAGAT
1448S-F	GAAGCATGCGCAACCATCAGACAAATTGC
1448-R1	AATGCATATCAGCGTGAGTCTTTC
1448L-R	GCGAAGCTTAATGCATATCAGCGTGAGTC
1449-F1	ATCTGTGTGAAATTGAGGGAATAC
1449S-F	GAAGCATGC ATCTGTGTGAAATTGAGGG
1449-R1	CTTCTCCATCATAAATTTCCCCAT
1449-R2	GTGCAATTTGTCTGATGGTTGC
1449L-R	GCGAAGCTTCTTCTCCATCATAAATTTCC
CAT-3	CGCGGATCCGATCGGCACGTAAGAGGTTC
CAT-4	CGCGGATCCCGTAGCACCAGGCGTTTAAG
HlyA-F	GAAGCATGCGTCTTTAGAGGCTAAAATCTG
HlyA-F9	GAGACACATGCAAAATGGGTATG
HlyA-R	GCGAAGCTTGCGCAACGATTGAGTTTTGG
HlyA-R7	GCGCAACGATTGAGTTTTGGCAT
HlyA2-F2	GGATATGCATTTCTGCTAAAAG
HlyA2-R2	GCGCAACGATTGAGTTTTGG
HlyU-F1	GCTCTAGATTTAGGATACATTTTTATGCCGTATTTAAAGGGGGC
HlyU-R1	ACATGCATGCCTAATGATGATGATGATGATGCTGATTCGCCTGACAATAAAG
HlyU-U1	GCCGAGCTCGTTCCAGGCAGTCGAACCG
HlyU-U2	AAAGGATCCCGGCATTTTAATTCCAACCC
HlyU-D3	GAAGGATCCGGCGAATCAGTAGGGTGGTC
HlyU-D4	TAAGCATGCCCGCGAAGGCTCTTCGATAC
RnnB-F1	CGGGGTACC GATTTTCAGCCTGATAC
RnnB-R1	CGCGGATCC TGGCTTGTAGATATGAC
RpoE-F	GAAGCATGCCGGGTGAAAATGCTGCCTTG
RpoE-R	GCGAAGCTTTCCTATTGTTATTCCCCTAC
RpoE-P2-F2	CGGGTGAAAATGCTGCCTTGAT
RpoE-P2-R2	TCCTATTGTTATTCCCCTACCTTC
RpsM-F51	GCAACTGCGTGATGGTGTAGCTAA
RpsM-R52	GCTTGATCGGCTTACGCGGACC
TcpA-F1	GTTCATAATTTCGATCTCCACTCCG
TcpA-R2	GTTAACCACACAAAGTCACCTGCAA
TnaA-F3	CCTGCGTAATCCCTTCTCGC
TnaA-R3	GTGTGGTGCGTTTAACTGG
TnaA-F	CGCGGATCCATGGGTTGGTCTCGCGCTG
Try-R	CTAGTCTAGAGTGTGGTGCGTTTAACTG
VC1922-F61	TAGAAGGTTGACGAAACAAGCAATCA
VC1922-R62	GGTTCAACCACCATAGGTACGAGT
VieA-U1	TGCTCTAGAGGGCTTTGAGCGCATGTTTG
VieA-U2	ACGCGTCGACAGAAATCGCCTTACAACTCG
VieA-D1	ACGCGTCGACCCATCCATCTGCGGCATTCG
VieA-D2	ACATGCATGCCTTATACCTTAGCCAATTTG
VieS-F	GAAGCATGCGTCCTGATCTTGGGCATC
VieS-R	GCGAAGCTTGTCCCACCCCAGTAAGGC
VieS-F2	GATATTGCTTGGGGTTGAAT
VieS-R2	TGTCCCACCCCAGTAAGGC
VieSAB-U1	TGCTCTAGATCAAAGCCTTACCGTGCATC
VieSAB-U2	ACGCGTCGACTCTCGCTACTGGCCGACG
VieSAB-D1	ACGCGTCGACCGCTTGCTCATTCGGATCCAAA
VieSAB-D2	ACATGCATGCGTAATTACACAGTATATTTCCC

### Construction of mutants

To construct *vieA* deletion mutants, we amplified DNA fragments flanking the *vieA* locus (VC1652) from C7258 genomic DNA using primer pairs VieA-U1/ VieA-U2 and VieA-D1/VieA-D2 (Table 2). The PCR products were sequentially cloned in pUC19 to yield pUCΔVieA and confirmed by DNA sequencing. The chromosomal DNA fragment harboring the *vieA* deletion was subcloned in the suicide vector pCVD442 [31] to yield pCVDΔVieA. This vector was transferred by conjugation to strains C7258ΔlacZ and AJB80 (Δ*hns*) (Table 1) and exconjugants were selected in LB agar containing Amp and PolB. Then, the *vieA* deletion mutants AJB606 and AJB607 were isolated by sucrose selection as previously described [25, 27, 32–34]. Similarly, for the construction of a *vieSAB* deletion mutant, we amplified DNA fragments flanking the entire *vieSAB* cluster (VC1651-VC1652-VC1653) using primer pairs VieSAB-U1/VieSAB-U2 and VieSAB-D1/VieSAB-D2 (Table 2). The PCR products were sequentially cloned in pUC19 to yield pUCΔvieSAB and the chromosomal DNA fragment harboring the *vieSAB* deletion was subsequently moved to pCVD442 to yield pCVDΔvieSAB. This plasmid was transferred by conjugation to strains C7258ΔlacZ and AJB80 and strains AJB600 and AJB601 harboring *vieSAB* deletions were isolated by sucrose selection. An identical strategy was used to construct *V*. *cholerae* Δ*hlyU* mutants, except that primer pairs HlyU-U1/HlyU-U2 and HlyU-D3/HlyU-D4 were used and the chloramphenicol acetyl transferase (*cat*) gene conferring chloramphenicol resistance was inserted in place of the deleted *hlyU* DNA. The *cat* gene was amplified with primers CAT-3 and CAT-4 from plasmid pCAT kindly provided by B. Kan (China CDC Beijing). The *hlyU* deletion/insertion was introduced into strains C7258Δ*lacZ* and AJB80 by conjugation as described above. Finally, the segregants AJB602 and AJB603, harboring the *hlyU* deletions and *cat* insertions, were isolated by sucrose selection. A mutant with a deletion of *crp* encoding the cAMP receptor protein (CRP) and *hns* (AJB604) was constructed by conjugal transfer of the suicide vector pCVDΔHNSK [27] to the *crp* mutant WL7258ΔlacZ [27] followed by sucrose selection. Similarly, a mutant lacking the quorum sensing regulator HapR and *hns* was constructed by conjugal transfer of pCVDΔHNSK to strain AJB51Δ*lacZ* resulting in strain AJB700. Finally, we constructed an *hns* mutant in a classical biotype background. To this end, we transferred the suicide vector pCVDΔHNSK [27] to strain O395Δ*lacZ* [35]. Exconjugants were selected in LB medium containing Amp and Str and strain AJB608 isolated as described above.

### Construction of *vieSAB*-, *tnaA*-, *hlyA*-, *rtxCA*-, *rtxBDE*-, and *rpoE*-*lacZ* promoter fusions

To construct a *vieSAB*-*lacZ* promoter fusion, we amplified a 633 bp fragment containing the *vieSAB* promoter of strain C7258Δ*lacZ* and O395Δ*lacZ* with primers VieS-F and VieS-R. For the *hlyA-lacZ* promoter fusion, a 540 bp fragment containing the *hlyA* promoter was amplified with primers HlyA-F and HlyA-R. A 250 bp *rtxCA* promoter fragment [36] was amplified with primers 1449S-F and 1449L-R. To construct the *rtxBDE-lacZ* promoter fusion, a 230 bp fragment containing the *rtxBDE* promoter [36] was amplified with primers 1448S-F and 1448L-R. For the *rpoE* ^P1^-*lacZ* and *rpoE* ^P2^-*lacZ* promoter fusions, a 190-bp fragment containing the *rpoE* P1 promoter and a 180-bp fragment containing the *rpoE* P2 promoter as described elsewhere [37] were amplified with primer combinations 70-F/70-R and RpoE-F/RpoE-R, respectively. The promoter fragments were inserted downstream of the *rrnB*T_1_T_2_ transcription terminator in plasmid pTT3 [38] to generate pTT3VieSAB^ET^, pTT3VieSAB^CL^, pTT3HlyA, pTT3RtxCA, pTT3RtxBDE, pTT3RpoE^P1^, and pTT3RpoE^P2^, respectively. Finally, the terminator-promoter fragments were inserted upstream of a promoterless *lacZ* gene in plasmid pKRZ1 [39] to generate pVieSAB-LacZ^ET^, pVieSAB-LacZ^CL^, pHlyA-LacZ, pRtxCA-LacZ, pRtxBDE-LacZ, pRpoE^P1^-LacZ and pRpoE^P2^-LacZ (Table 1). To construct a *tnaA-lacZ* promoter fusion, we first amplified the *rrnB*T_1_T_2_ transcription terminator from plasmid pTT3 with primers RnnB-F1and RnnB-R1 and re-inserted it in pUC19 to generate pTT5. Then, a 1140 bp fragment containing the *tnaA* promoter and transcribed leader region [40] was amplified with primers TnaA-F and Try-R. The promoter fragment was inserted downstream of the *rrnB*T_1_T_2_ transcription terminator in plasmid pTT5 to generate pTT5TnaA. Finally, the terminator-promoter cassette was inserted upstream of a promoterless *lacZ* gene in plasmid pKRZ1 to generate pTnaA-LacZ. The construction of plasmids pCTX-lacZ containing a *ctxA-lacZ* promoter fusion and pRpoE-LacZ containing a both *rpoE* P1 and P2 promoters ligated to a promoterless *lacZ* gene has been described previously [35]. The *lacZ* promoter fusions were introduced into strains C7258Δ*lacZ* (WT), AJB80 (Δ*hns*), AJB602 (Δ*hlyU*), AJB603 (Δ*hns*Δ*hlyU*), WL7258ΔlacZ (Δ*crp*), AJB604 (Δ*crp*Δ*hns*) AJB606 (Δ*vieA*), AJB607 (Δ*hns*Δ*vieA*), AJB600 (Δ*vieSAB*), AJB601 (Δ*hns*Δ*vieSAB*), O395Δ*lacZ*, and AJB608 (Δ*hns*) by electroporation.

### Total RNA extraction and removal of ribosomal RNA

**S**trains C7258ΔlacZ and AJB80 were grown in 50 mL of LB medium at 37°C with agitation to optical densities at 600 nm (OD_600_) of 0.5 and 2.0. Each culture was divided into 5 mL aliquots and the cells were harvested by centrifugation at 4,000 × g for 10 min at room temperature. The pellets were resuspended in 5 mL of RNAlater (Invitrogen) and agitated on a rotator for 10 min at room temperature. The cells were collected by centrifugation at 4,000 × g for 10 min and resuspended in 5 mL of RNAlater. Then, cell pellets corresponding to 1 mL aliquots were collected by centrifugation for 10 min at 4,000 × g. Total RNA was extracted using RNeasy Plus Mini Kit (Qiagen). To this end, the cell pellets were resuspended in 200 μL of bacterial lysis buffer [30 mM Tris-HCl pH 8.0, 1 mM EDTA, 15 mg/mL lysozyme (Sigma-Aldrich)], supplemented with 15 μL of proteinase K (20 mg/mL, Qiagen), and the samples were incubated at room temperature for 10 min with vortexing every 2 min. Qiagen RLT Plus buffer (750 μL) containing 1% v/v 2-mercaptoethanol was added to each sample and the tubes were briefly vortexed. The bacterial lysates were homogenized using QIAshredder spin columns (Qiagen) and genomic DNA removed using gDNA Eliminator spin columns (Qiagen). Finally, total RNA was recovered using the RNeasy spin column method (Qiagen) following the manufacturer’s protocol. RNA integrity was determined by formaldehyde agarose gel electrophoresis and the RNA was stored at -80°C. Contamination with DNA was further eliminated using the TURBO DNA-free kit (Invitrogen), which involves a second treatment with DNase for 30 min at 37°C. Reactions were terminated by addition of 0.2 volumes of the DNase inactivation reagent and RNA was purified using the Agencourt RNAClean XP kit (Beckman) following the manufacturer’s instructions. Total RNA was eluted in 60 μL of RNase-free water. The absence of DNA contamination was confirmed by PCR with 16S-specific primers 357F and 926R. The DNase treated RNA (2 μL) was added to each reaction in a final reaction volume of 20 μL. Each reaction was run in parallel to *E*. *coli* DNA (positive control) and nuclease-free water (negative control) with the following cycling conditions: 95°C for 1 min, 30 cycles of 95°C for 30 s, 50°C for 30 s, 68°C for 1 min. Next, rRNA was removed using Ribo-Zero Magnetic Kit (Epicentre) following the manufacturer’s instructions. Briefly, 6 μg of total RNA sample and 20 μL of Ribo-Zero rRNA Removal Solution were combined in a final reaction volume of 80 μL. Samples in Ribo-Zero rRNA removal solution were incubated at 68°C for 10 min followed by a 5 min incubation at room temperature. To remove the rRNA molecules from the mRNA, reactions mixtures were incubated with the magnetic beads provided in the kit, mixed and placed at room temperature for 5 min and then at 50°C for 5 min. The rRNA bound to the beads was then removed by magnetic separation. Finally, mRNA was purified using Agencourt RNAClean XP kit and eluted in 15 μL of RNase-free water.

### RNA-seq and data analysis

RNA-seq was performed as described elsewhere [41] on the Illumina HiSeq2000 using the latest versions of sequencing reagents and flow cells providing up to 300 Gb of sequence information per cell. Briefly, the quality of the total RNA was assessed using the Agilent 2100 Bioanalyzer and samples were subsequently converted to cDNA. Libraries were constructed using the TruSeq library generation kits as per the manufacturer’s instructions (Illumina, San Diego, CA). The cDNA libraries were quantitated using qPCR in a Roche LightCycler 480 with the Kapa Biosystems kit for library quantitation (Kapa Biosystems, Woburn, MA) prior to cluster generation. Clusters were generated to yield approximately 725K—825K clusters/mm^2^. Paired end 2X50 bp sequencing runs were conducted to align the cDNA sequences to the reference genome. The TopHat software and the short read aligner Bowtie was used to align the raw RNA-seq fastq reads to the reference genome [42–44]. Transcripts assembly, abundance and evaluation of differential expression and regulation were accomplished using the Cufflinks software [42, 43]. Genes exhibiting a fold change ≥ ±2.0 and q-value < 0.05) were considered differentially expressed in the Δ*hns* mutant.

### Protein-DNA interaction assays

**Electrophoresis mobility shift assay**. Electrophoresis mobility shift assays (EMSA) were conducted using the second-generation digoxigenin (DIG) gel shift kit (Roche) as previously described [25, 32, 34]. Protein-DNA complexes were separated by electrophoresis in 5% Tris-borate-EDTA (TBE) polyacrylamide gels and transferred to nylon membranes, and DNA was visualized using an anti-DIG Fab fragment-AP conjugate, followed by chemiluminescence detection. The following primer combinations were used to amplify the promoter regions under study: VieS-F2 and VieS-R2 for *vieSAB*, TnaA-F3 and TnaA-R3 for *tnaA*, 1448-F1 and 1448-R1 for *rtxBDE*, 1449-F1 and 1449-R1 for *rtxCA*, HlyA2-F2 and HlyA2-R2 for *hlyA*, 70-P1-F1 and 70-P1-R1 for *rpoE* P1, RpoE-P2-F2 and RpoE-P2-R2 for *rpoE* P2, VC1922-F61 and VC1922-F62 for the negative control VC1922, and TcpA-F1 and TcpA-R2 for the positive control *tcpA*.

**Chromatin immunoprecipitation**. Occupation of the *vieSAB*, *tnaA*, *rtx* and *hlyA* promoters by H-NS in the cell was determined by chromatin immunoprecipitation (ChIP). To this end, strain C7258HNS-FLAG expressing an *hns*-FLAG allele from native transcription and translation signals [25, 32] was grown to an OD_600_ of 0.5. Nucleoprotein complexes were immunoprecipitated (IP) with the anti-FLAG M2 monoclonal antibody (mAb) (Sigma-Aldrich) as previously described [25, 32, 34]. IP DNA was qualitatively detected by PCR and agarose gel electrophoresis using the PCR primers used to generate promoter fragments for EMSA. Real-time quantitative PCR (qPCR) was used to quantitate promoter occupancy by H-NS-FLAG as formerly described [25, 32, 34]. The quantity of IP DNA was calculated as a percentage of the input DNA (10 μL sample taken prior to IP) using the formula IP = 2^(CT^_input_^-CT^_IP_^)^, where CT is the fractional threshold cycle of the input and IP DNAs. The relative IP was calculated by normalizing the IP of each sample by the IP of a mock ChIP using the unrelated mouse monoclonal antibody G3A1 IgG1 isotype control (Cell Signaling Technology). To study the effect of HlyU on H-NS occupancy at the *hlyA* and *rtx* promoters strain C7258HNS-FLAG containing pBAD-HlyU-His was grown in LB medium containing Amp and Cm at 37°C with agitation to OD_600_ 0.5. At this point the cultures were divided in halves, with one half used as a control and the other induced by addition of L-arabinose to a final concentration of 0.02%. The cultures were incubated for 3 h and the cells collected and processed for ChIP as described previously [25, 32, 34]. For this experiment, a region of the *hlyA* promoter containing putative H-NS binding sites was amplified with primers HlyA-F9 and HlyA-R7. The *rtxCA* and *rtxBDE* promoter regions were amplified with primer combinations 1449-R1/1449-R2 and 1448-F2/1448-R1, respectively. The *tcpA* promoter, which is not regulated by HlyU, was used as a positive control for H-NS occupancy and amplified with primers TcpA-F1 and TcpA-R2. Negative controls consisted of the VC1922 promoter amplified with primers VC1922-F61 and VC1922-R62 and a region within the *rpsM* open reading frame (ORF) amplified with primers RpsM-F51 and RpsM-R52.

### Cloning and overexpression of the regulator HlyU

The *hlyU* open reading frame (ORF) was amplified from strain C7258 genomic DNA with primer HlyU-F1 and HlyU-R1 incorporating a C-terminal 6xHis tag. The PCR product was ligated into pCR2.1 and confirmed by DNA sequencing. Then, a 373 bp *Xba*I-*Sph*I DNA fragment from the resulting plasmid was subcloned into pBAD33 [45] to generate pBAD-HlyU-HIS. Finally, pBAD-HlyU-HIS was transformed into C7258HNS-FLAG by electroporation. Expression of the HlyU-6xHis protein was confirmed by western blot using the histidine tag monoclonal antibody 3D5 (Life Technologies).

### Measurement of indole, hemolysin activity and biofilm formation

Indole production was measured as described in [46]. To this end, 0.4 mL of cell-free culture supernatant was treated with the same volume of 20% trichloroacetic acid, mixed, incubated 10 min on ice and cleared by centrifugation. Then 0.4 mL of the treated supernatant was reacted with the same volume of Kovac’s reagent (Sigma-Aldrich). Indole production was estimated using a standard curve of commercial indole (Sigma-Aldrich) and reported as μM indole normalized by the culture OD_600_. Hemolysin activity on sheep red blood cells was conducted as described in [47] except that a unit of enzyme activity was defined as the amount of enzyme causing an increase in 0.01 optical density units at 570 nm per h at 37°C. Biofilm formation was measured by the crystal violet staining method and expressed as optical density at 570 nM [33, 34].

### Enzyme activities

β-Galactosidase activity was measured as described in [48] using the substrate o-nitro phenyl-β-D-galactopyranoside. Specific activities are given in Miller units [1,000 × (OD420/*t* ×*v* ×OD600)], where *t* is the reaction time and *v* is the volume of enzyme extract per reaction.

### Chemotaxis capillary assay

A capillary chemotaxis assay was conducted as described previously [49]. Briefly, overnight cultures of *V*. *cholerae* strains in TG medium were diluted 1:30 in fresh medium and incubated with agitation for 6 h at 30°C. The cells were collected by centrifugation and washed with TM buffer consisting of 50 mM Tris-HCl pH 7.4, 5 mM glucose and 5 mM MgCl_2_. Next, the cells were resuspended in TMN buffer (50 mM Tris-HCl pH 7.4, 5 mM glucose, 100 mM NaCl and 5 mM MgCl_2_) to OD_600_ 0.1. Cells were pre-incubated for 1 h at 30°C and a capillary containing L-glycine (10 mM), L-serine (10 mM) or water inserted. The capillary was incubated in the cell suspension 1 h at 30°C after which, the number of cells within the capillary was determined by plating serial dilutions on LB agar.

### Infant mouse colonization assay

A competitive infant mouse colonization assay was conducted using 3–5 days old CD-1 newborn mice as previously described [33]. The study was conducted in strict accordance with the recommendations in the Guide for the Care and Use of Laboratory Animals of the National Institutes of Health and the protocol approved by the Morehouse School of Medicine Institutional Animal Care and Use Committee (protocol # 13–27). A competitive index (CI) was calculated by normalizing the mutant to wild type ratio after intestinal colonization (output) by the mutant to wild type ratio in the inoculum (input).

### Statistical evaluation

Differences between the means of measured activities obtained for different strains, mutants, transformants and culture conditions were evaluated for statistical significance using an unpaired, one-tailed t test and p values are cited in the text when required.

## Results

### Transcription profile of a *V*. *cholerae* El Tor biotype Δ*hns* mutant

We compared the transcription profile of a wild type *V*. *cholerae* strain of the El Tor biotype and its isogenic *hns* mutant grown in LB medium by RNA-seq. El Tor biotype *hns* mutants are known to express major virulence factors under this culture condition [27, 28]. A complete list of genes differentially expressed in the *hns* mutant and their distribution per role category is provided as supporting information in S1 Fig., S1 Table and S2 Table. The raw and processed datasets have been deposited in the Gene Expression Omnibus (GEO) repository (http://www.ncbi.nlm.nih.gov/geo/) and assigned accession number GSE62785.

**Fig 1:**
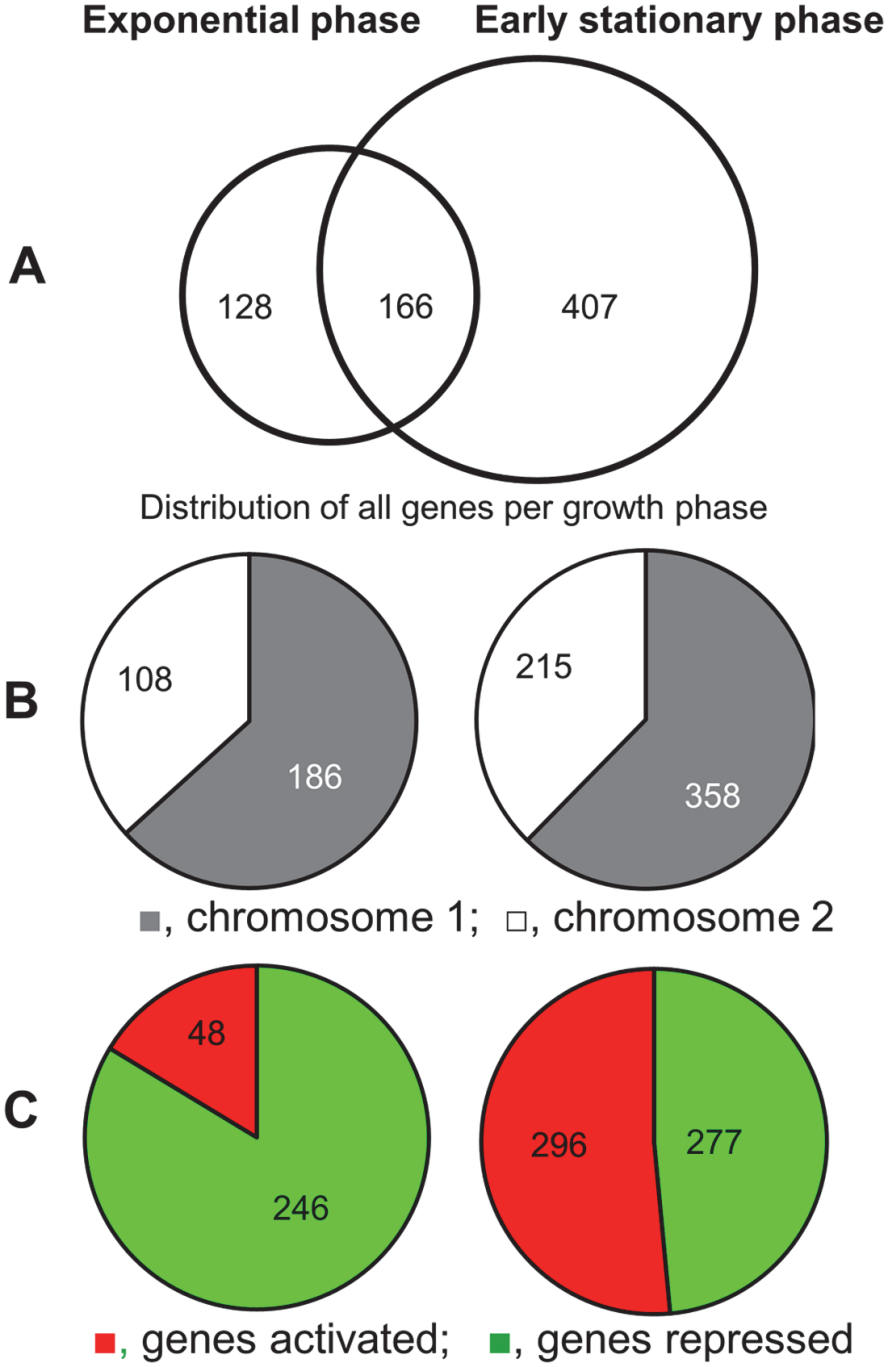
Venn diagram representation of the number of genes differentially expressed in an *hns* mutant. Left side, data from exponential phase; right side, data from early stationary phase. **A**. Number of H-NS regulated genes in exponential and early stationary phases. **B**. Distribution of H-NS regulated genes per chromosome in exponential and early stationary phases. **C**. Distribution of genes positively or negatively regulated by H-NS in exponential and early stationary phases. The data shown resulted from the analysis of two independent cultures of each strain collected in exponential phase and two cultures collected in early stationary phase.

We identified 701 genes differentially expressed in the Δ*hns* mutant defined as exhibiting a fold change ≥ ±2.0 and q-value < 0.05 (Fig. 1A). As expected, genes known to be transcriptionally silenced by H-NS such as *ctxAB*, the *tcpA-F* cluster, *tcpPH*, *toxT* [23, 24, 28, 50] and *vps* genes required for biofilm matrix exopolysaccharide [32] were identified in the dataset. Most H-NS regulated genes fell within the role categories of cellular processes, energy metabolism, regulatory function, and transport and binding proteins (S1 Fig.). The number of differentially regulated genes located in chromosomes I and II exhibited no bias and correlated with chromosome size (Fig. 1B).

The *hns* transcriptome differed markedly in exponential and early stationary phases. For instance, the number of genes identified to be regulated by H-NS was higher in the early stationary phase (573) compared to exponential phase (294). Further, H-NS appeared to function mainly as a transcriptional repressor in exponential phase (Fig. 1C), while a larger fraction of genes were found to be positively regulated by H-NS in early stationary phase (Fig. 1C). A selection of genes newly identified to be regulated by H-NS and affecting virulence, stress response, chemotaxis and biofilm development is shown in Table 3.

**Table 3:** Selected new genes regulated by H-NS affecting *V. cholerae* virulence, stress response and chemotaxis.

**Loci**	**Genes**	**FC** *	**Loci**	**Genes**	**FC** *
**Exponential phase**			**Early stationary phase**		
**Virulence**					
VC1447, VC1448	*rtxD*, *rtxB*	3.1	VC1447, VC1448	*rtxD*, *rtxB*	2.5
			VC1449, VC1450	*rtxC*, *rtxA*	4.7
VC1651	*vieB*	2.7	VC1651	*vieB*	1.5
VC1652	*vieA*	2.9	VC1652	*vieA*	1.8
VC1653	*vieS*	3.1	VC1653	*vieS*	2.2
VCA0219	*hlyA*	5.2	VCA0219	*hlyA*	1.7
**Cell envelope, transport and binding proteins, stress response**					
VC0156	*tonB*	-2.4	VC0364	*Bfd*	1.2
VC1318	*ompV*	1.2	VC0365	*Bfr*	-1.4
VC1565	*tolC*	2.1	VC0773	*vibC*	-1.0
VC1583	*sodC*	2.9	VC1565	*tolC*	0.9
VC1585	*katB*	3.7	VC1854	*ompT*	-5.6
VC2464,VC2466, VC2465	*rseA*, *rseB*, *rseC*	1.8	VC2213	*ompA*	1.6
VC2467	*rpoE*	2.1	VCA0576	*hutA*	2.5
VCA0576	*hutA*	2.6	VCA0867	*ompW*	-1.1
VCA0915	*hutD*	1.4	VCA1028	*ompS*	1.1
VCA1028	*ompS*	3.4	VC1583	*sodC*	3.5
			VC1585	*katB*	5.7

Loci	Gene or annotation	FC
**Chemotaxis and motility**		
**Exponential phase**		
VC1313	MCP**	-1.4
VCA0220	MCP	11.8
VCA1069	MCP	11.4
**Early stationary phase**		
VC1967	MCP	-1.0
VC0098	MCP	-1.2
VC1248	MCP	-2.1
VC1298	MCP	-0.9
VC1313	MCP	-0.8
VC1394	MCP	-1.2
VC1401	*cheB*	-1.0
VC1402	*cheW*	-1.5
VC1403	MCP	-1.4
VC1868	MCP	-1.4
VC2064	*cheZ*	-0.9
VCA0008	MCP	-1.4
VCA0031	MCP	-1.9
VCA0268	MCP	-1.5
VCA0864	MCP	-0.8
VCA0906	MCP	-1.3
VCA0923	MCP	-1.0
VCA0979	MCP	-2.1
VCA0988	MCP	-0.9
VCA1034	MCP	-1.5
VCA1056	MCP	-1.1
VCA1088	MCP	-1.3
VCA1090,VCA1091	*cheD*, *cheR*	-1.7
VCA1092	MCP	-1.4
VCA1093,VCA1094	*cheW-2*, *cheW-3*	-1.5
VCA1095	*cheA-3*	-1.4
VCA1096,VCA1097	*cheY-4*	-1.3
**Cell-to-cell communication and biofilm development**		
**Exponential phase**		
VCA0160	*tnaB*	3.2
VCA0161	*tnaA*	6.4

* Log_2_ fold change

** Methyl-accepting chemotaxis protein

### H-NS and quorum sensing silence the transcription of the *vieSAB* regulatory system

**Fig 2:**
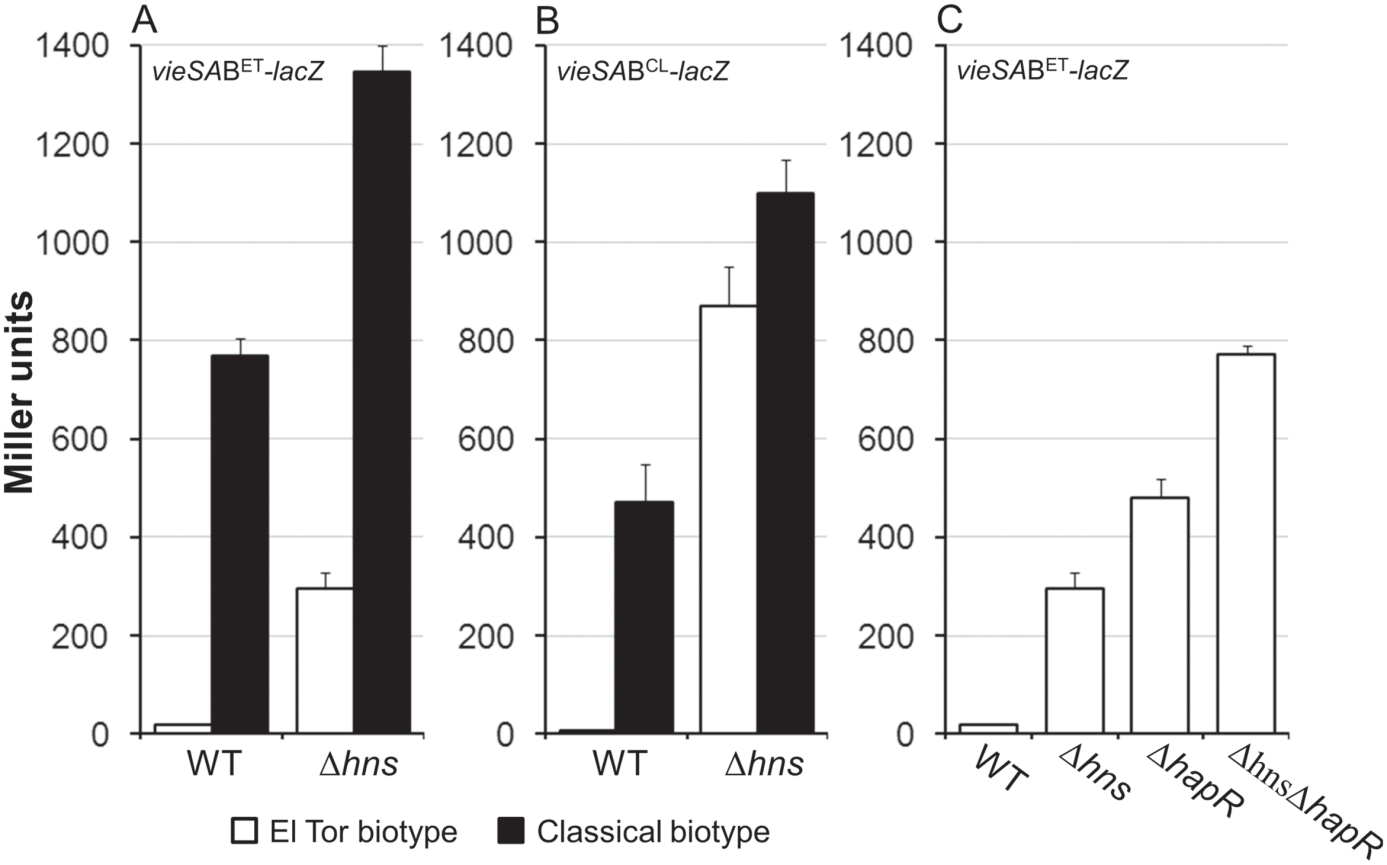
Repression of the *vieSAB* operon by H-NS and quorum sensing. **A**. β-galactosidase activity produced by wild type (WT) and mutant strains containing an El Tor biotype *vieSAB* ^ET^ *-lacZ* promoter fusion. El Tor biotype strain C7258Δ*lacZ* and classical biotype strain O395Δ*lacZ* and their isogenic Δ*hns* mutants (AJB80 and AJB608, respectively) were grown to stationary phase in LB medium. **B**. β-galactosidase activity produced by wild type (WT) and mutant strains containing a classical biotype *vieSAB* ^CL^ *-lacZ* promoter fusion. Strains C7258Δ*lacZ* and O395Δ*lacZ* and their isogenic Δ*hns* mutants were grown to stationary phase in LB medium. **C**. Activity of the *vieSAB* ^ET^ *-lacZ* promoter fusion in wild type (C7258Δ*lacZ*), Δ*hns* (AJB80), Δ*hapR* (AJB51Δ*lacZ*) and Δ*hns*Δ*hapR* (AJB700) mutants grown to stationary phase in LB medium. β-galactosidase activity (Miller units) was measured as an indicator of promoter activity. Each value represents the mean of six independent experiments. Error bars denote the standard deviation (STDEV).

The *vieSAB* operon encodes a three component regulatory system that enhances the expression of *ctxAB in vitro* and during infection [51–53]. The El Tor biotype *hns* mutants expressed elevated *vieSAB* (Table 3). To confirm this result, we constructed *vieSAB*-*lacZ* fusions containing the El Tor biotype *vieSAB* promoter (*vieSAB* ^ET^) promoter connected to a promoterless *lacZ* gene. In Fig. 2A we show that the El Tor biotype *hns* mutant AJB80 grown in LB medium expressed 17-fold higher *vieSAB* ^ET^ *-lacZ* promoter activity compared to wild type (p < 0.001). We constructed an *hns* mutant in the classical biotype strain O395Δ*lacZ* and measured the expression of the *vieSAB* ^ET^ *-lacZ* promoter fusion in wild type and mutant. As shown in Fig. 2A, the wild type classical biotype strain expressed 54-fold higher *vieSAB* ^ET^ promoter activity compared to the El Tor biotype strain. Further, deletion of *hns* in the classical biotype resulted in a modest 1.7-fold increase in *vieSAB* ^ET^-*lacZ* activity suggesting that H-NS is 10 times less effective in repressing the same promoter when it is introduced in the classical biotype genetic background. A similar experiment was conducted using the *vieSAB* ^CL^ promoter. As shown in Fig. 2B, the *vieSAB* ^CL^ promoter was also silent in the wild type El Tor background and significantly derepressed in the El Tor *hns* mutant. We note that the *vieSAB* ^CL^ promoter activity was higher in the El Tor *hns* mutant compared to the *vieSAB* ^ET^ promoter in the same genetic background. The reverse trend was observed for the *vieSAB* ^CL^ promoter in wild type El Tor and classical biotype strains. In spite of these differences, our data shows that both promoters are transcriptionally silenced by H-NS in the El Tor biotype and are expressed in the wild type classical biotype background in which H-NS appears to function as a weaker repressor. The classical biotype strain used in our study is known to lack the quorum sensing regulator HapR. Thus, we considered the possibility of *vieSAB* being silenced in the El Tor biotype by quorum sensing in addition to H-NS. In Fig. 2C we show that deletion of *hapR* in strain C7258Δ*lacZ* resulted in significant derepression of *vieSAB* and that the Δ*hns*Δ*hapR* double mutant exhibited the highest (approximately additive) *vieSAB* promoter activity. We conclude that both, H-NS and quorum sensing contribute to the transcriptional silencing of *vieSAB* in El Tor biotype *V*. *cholerae*.

**Fig 3:**
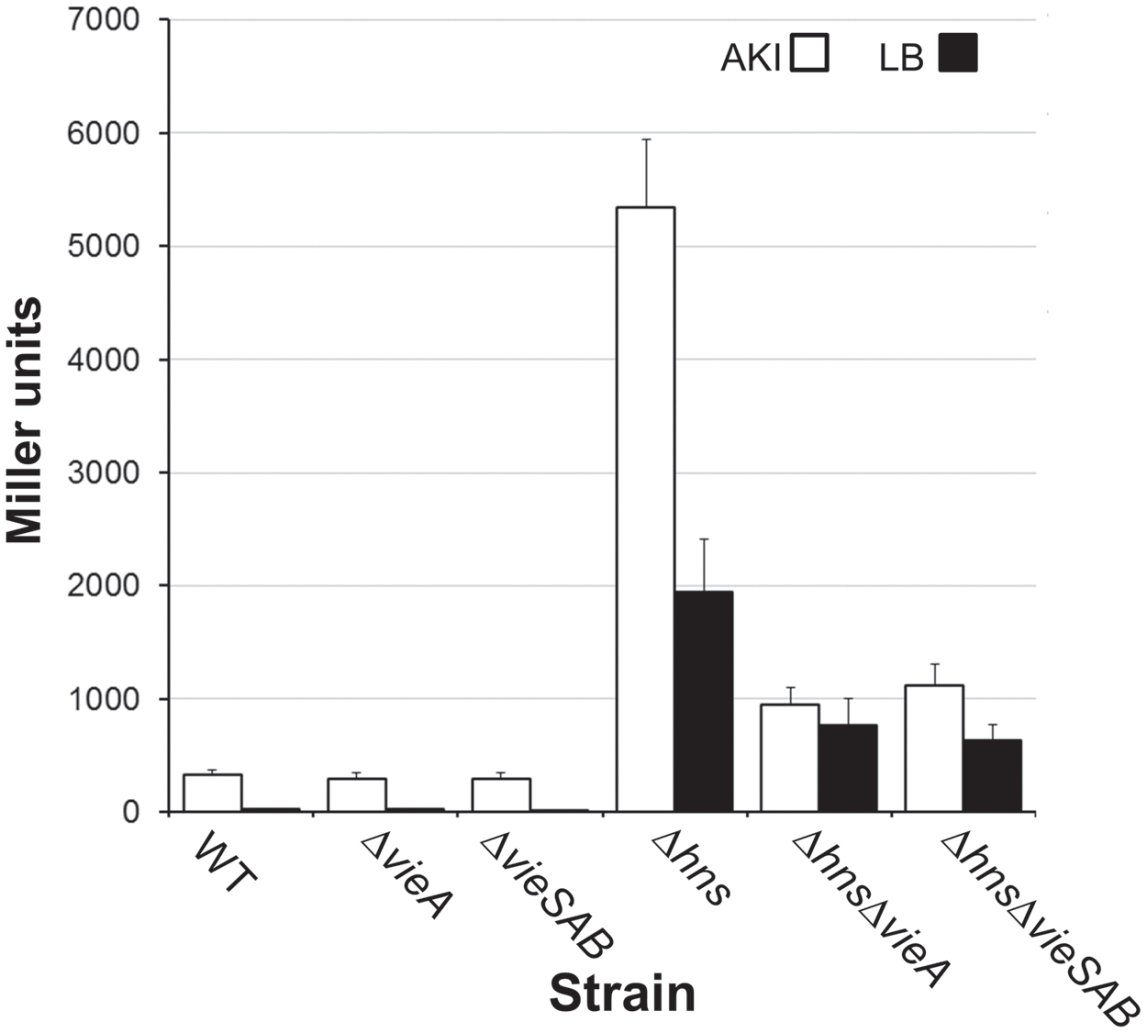
Expression of *ctxA* in wild type, *vieA*, *vieSAB* and *hns* mutants. El Tor biotype WT and mutant strains containing a *ctxA-lacZ* promoter fusion were grown in ToxR-permissive AKI medium and non-permisive LB medium. β-galactosidase activity (Miller units) was measured as an indicator of promoter activity. Each value represents the mean of six independent experiments. Error bars denote the standard deviation (STDEV).

Since VieA has been suggested to enhance CT production [51–53], we examined if derepression of VieA contributed to the expression of CT genes under ToxR non-permissive conditions in the *hns* mutant. To this end, we constructed a *ctxA*-*lacZ* promoter fusion and compared its activity in El Tor biotype wild type, *vieA*, *vieSAB* and *hns* mutants grown in ToxR non-permissive LB and AKI media. Deletion of *vieA* and/or *vieSAB* in the wild type genetic background did not affect *ctxAB* expression under both culture conditions consistent with *vieSAB* being transcriptionally silenced by H-NS and quorum sensing (Fig. 3). However, deletion of *vieA* and/or *vieSAB* significantly diminished *ctxA*-*lacZ* expression in the *hns* genetic background under both culture conditions (Fig. 3, p < 0.001).

### Regulation of the *hlyA* and *rtx* promoters by H-NS and HlyU

El Tor biotype strains have been shown to express additional toxic factors such as hemolysin and the RTX toxin [1, 2]. As shown in Table 3, the Δ*hns* mutant overexpressed *hlyA* encoding hemolysin and genes required for the biosynthesis (*rtxCA*) and transport (*rtxBDE*) of the RTX toxin.

**Fig 4:**
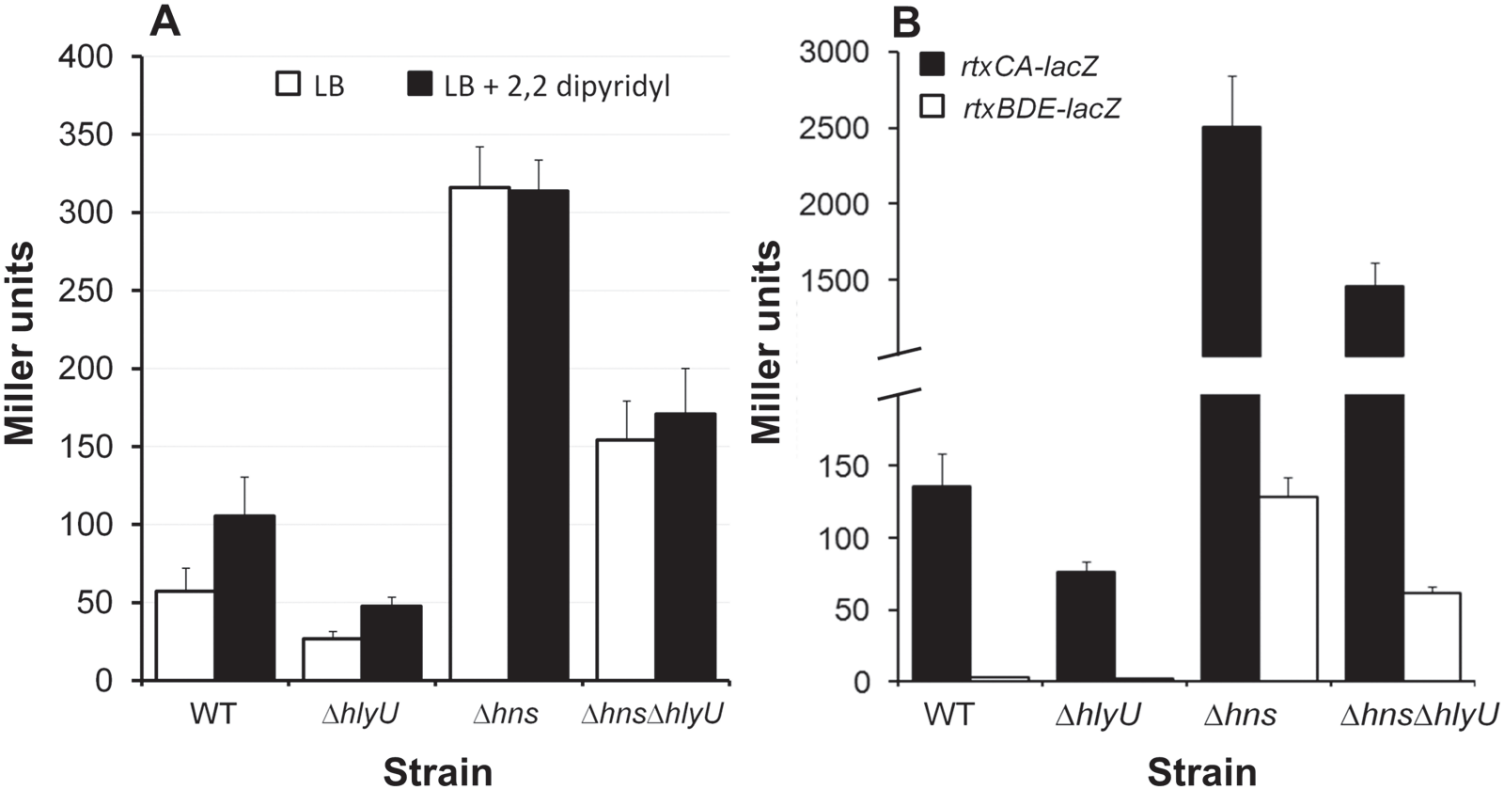
H-NS repression of hemolysin and RTX toxin expression. **A**. β-galactosidase activity produced by wild type (WT) and mutant strains containing a *hlyA-lacZ* promoter fusion. WT and mutants were grown in LB to an OD_600_ 0.5. The cultures were divided in halves, one half treated with 2,2 dipyridyl for 2 h at 37°C and the other half used as a control. **B**. β-galactosidase activity produced by wild type (WT) and mutant strains containing a divergently transcribed *rtxAC*- and *rtxBDE-lacZ* promoter fusions. WT and mutants were grown in LB to stationary phase at 37°C. β-galactosidase activity (Miller units) was measured as an indicator of promoter activity. Each value represents the mean of six independent experiments. Error bars denote the STDEV.

**Hemolysin expression**. Very little hemolysin activity could be detected in exponential phase culture supernatants (OD_600_ 0.6) in the wild type strain (1.5 ± 0.96 units/OD_600_) compared to the Δ*hns* mutant (11.5 ± 5.4 units/OD_600_). In both cases, the hemolysin activity declined as the culture progressed to stationary phase (wild type, 0.1 ± 0.05 units/OD_600_; Δ*hns* 0.54 ± 0.14 units/OD_600_). Expression of *hlyA* in *V*. *cholerae* is regulated by iron [47] and HlyU, a member of the SmtB/ArsR family of metalloregulators [54]. To determine if this pattern of regulation was disrupted in the Δ*hns* mutant, we constructed an *hlyA-lacZ* promoter fusion. Wild type and mutant cultures containing the *hlyA-lacZ* fusion were grown in LB medium to mid-exponential phase and 2, 2,-dipyridyl was added to induce iron-limitation. In Fig. 4A we show that expression the *hlyA*-*lacZ* fusion is strongly enhanced in the Δ*hns* mutant (p < 0.01) and diminished in the Δ*hlyU* genetic background (p < 0.05). We note that deletion of *hlyU* still diminished *hlyA* expression in the absence of H-NS (Fig. 4A) (p < 0.05). However, the Δ*hns*Δ*hlyU* double mutant still expressed higher activity compared to wild type and hlyU strains. Expression of *hlyA* was significantly enhanced (p < 0.05) by inducing iron limitation with 2, 2,-dipyridyl in the wild type strain but not in the Δ*hns* mutants (Fig. 4A).

**Expression of the RTX toxin and transport system**. To confirm the overexpression of RTX toxin and transport genes (Table 3) we constructed *rtxCA-* and *rtxBDE-lacZ* promoter fusions. The transcription factor HlyU has been suggested to function as an H-NS anti-repressor of RTX toxin expression in *V*. *vulnificus* [55]. In Fig. 4B we show that expression of the divergently transcribed *rtxCA* and *rtxBDE* operons are enhanced in the Δ*hns* mutant and significantly diminished in the Δ*hlyU* genetic background (p < 0.01). Again, HlyU was still required for maximal expression of *rtxCA* and *rtxBDE* in the absence of H-NS (Fig. 4B).

### H-NS is a repressor of indole biosynthesis

**Fig 5:**
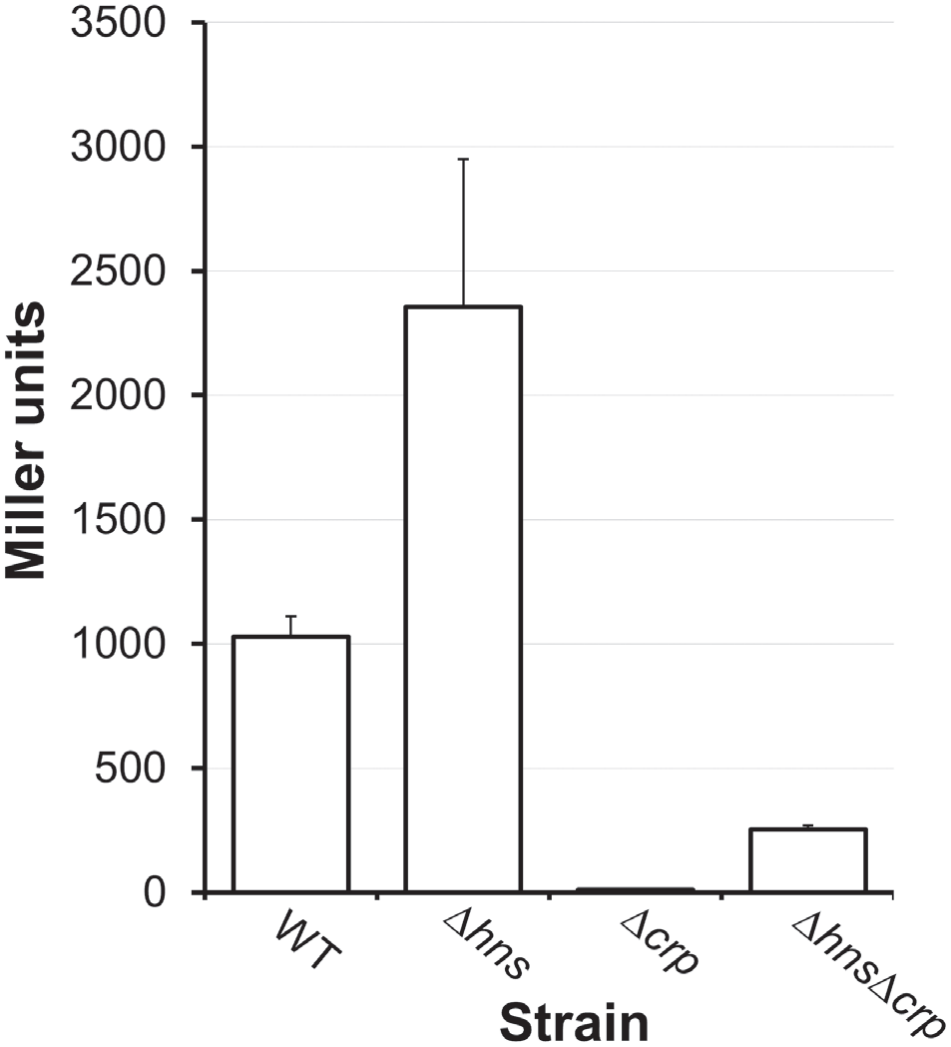
Deletion of *hns* enhances tryptophanase expression. WT and mutants containing a *tnaA-lacZ* promoter fusion were grown in LB to stationary phase at 37°C. β-galactosidase activity (Miller units) was measured as an indicator of promoter activity. Each value represents the mean of at least three independent experiments. Error bars denote the STDEV.

In the cell-to-cell communication catergory, the *hns* mutant expressed elevated *tnaA* (encoding tryptophanase) and tryptophan-transport gene VCA0160 in exponential phase (Table 3). Tryptophanase catalyzes the reductive deamination of tryptophan to indole. Indole has been reported to act as a signaling molecule in *V*. *cholerae* that enhances biofilm development [46]. We found that the Δ*hns* mutant accumulated 317 ± 26 μM of indole in the medium compared to 160 ± 26 in the wild type precursor (n = 3). We confirmed that growth of the wild type strain C7258 in LB supplemented with exogenous indole (300 μM) resulted in a 4-fold increase in biofilm formation (OD_570_ in LB 0.62 ± 0.05; OD_570_ LB + indole 2.45 ± 0.24; n = 3). In a previous microarray study, we showed that expression of *tnaA* is diminished in strain C7258 lacking CRP [56]. To examine the regulation of *tnaA* transcription in the *V*. *cholerae* Δ*hns* mutant, and the interplay between H-NS and CRP in the control of this promoter, we constructed a *tnaA*-*lacZ* promoter fusion. In Fig. 5 we show that *tnaA*-*lacZ* activity was enhanced in the Δ*hns* mutant (p < 0.05) and significantly diminished in an isogenic Δ*crp* mutant (p < 0.01). CRP was still required for full *tnaA* expression in the mutant lacking H-NS (Fig. 5).

### Mutational loss of *hns* induces an endogenous envelope stress response

**Fig 6:**
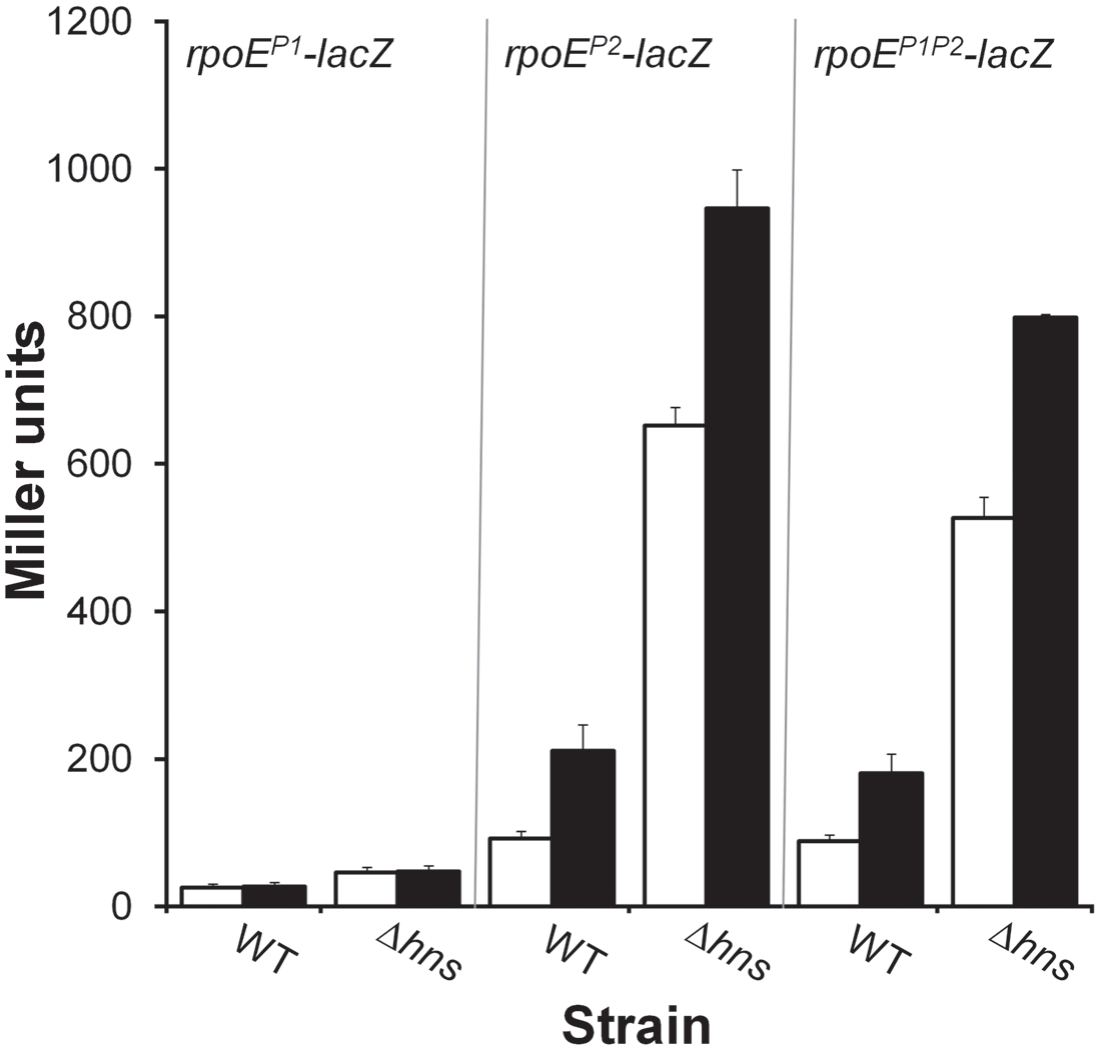
*hns* mutants express elevated levels of σ^E^ encoded by *rpoE*. WT and mutants containing different *rpoE*-*lacZ* promoter fusions described in materials and methods were grown in LB to stationary phase at 37°C in the presence and absence of PolB. β-galactosidase activity (Miller units) was measured as an indicator of promoter activity. The P1 fusion contained the *rpoE* σ^70^-dependent promoter; P2 contained the σ^E^-dependent promoter and the P1P2 fusion contained both promoters. Symbols: □, control; ■, PolB treated. Each value represents the mean of three independent experiments. Error bars denote the STDEV.

The *hns* mutant exhibited significant dysregulation of genes encoding outer membrane proteins (*ompA*, *ompS*, *ompT*, *ompV*, *ompW* and *tolC*) (Table 3). Some of these *omp* genes were overexpressed in the mutant (*ompA*, *ompS*, *ompV*, *tolC*), while *ompT* and *ompW* exhibited lower expression (Table 3). The mutant also exhibited aberrant expression of genes involved in haemin and iron binding and transport. For instance, *tonB* (haemin, vibriobactin and ferrichrome transport), *hutA* and *hutD* (haemin transport) and *bfd* (bacterioferritin-associated ferredoxin) were overexpressed while *bfr* (bacterioferritin) and *vibC* (vibriobactin) were down-regulated in the Δ*hns* genetic background (Table 3). We note that genes involved in iron and haemin (*bfd*, *bfr*, *vibC*, *hutA*) transport, as well as those encoding the major OMPs *ompT*, *ompA*, and *ompW*, were differentially expressed in early stationary phase only. The genes *sodC* (superoxide dismutase) and *katB* (catalase), involved in oxidative stress response, were overexpressed in both growth phases studied. In Table 3 we show that the *hns* mutant expressed elevated *rpoE* encoding the extra cytoplasmic RNA polymerase alternative sigma factor E (σ^E^) in exponential phase as well as genes *rseA*, *rseB*, *rseC* encoding regulators of *rpoE* activity (Table 3). Perturbation of the cell envelope has been reported to induce oxidative stress and changes in iron homeostasis [57]. Thus, our results suggested that H-NS plays a critical role in the biogenesis of the cell envelope. We hypothesized that mutational loss of *hns* could induce an endogenous envelope stress response resulting in the elevated transcription of *rpoE* in the absence of exogenous stressors. The *rpoE* gene is transcribed from two promoters; an upstream σ^70^-dependent promoter (P1) and an σ^E^-dependent promoter (P2) [37]. Upon the occurrence of an envelope stress, σ^E^ is released from the cell membrane and activates its own promoter (P2) [58, 59]. To test the hypothesis that loss of *hns* induces an endogenous envelope stress response, we constructed several promoter fusions containing the P1, P2 and both *rpoE* promoters fused to a promoterless *lacZ* gene. Then, the activity of each promoter fusion was tested in the absence and presence of an exogenous envelope stress induced by PolB. As expected, the σ^70^-dependent P1 promoter was not responsive to PolB (Fig. 6). In contrast, both the P2 and P1P2 *lacZ* promoter fusions were responsive to PolB and were significantly enhanced (p < 0.01) in the Δ*hns* mutant (Fig. 6). It is noteworthy that mutational loss of H-NS resulted in a much stronger induction of *rpoE*-*lacZ* activity compared to treatment with PolB. Treatment of the *hns* mutant with PolB enhanced *rpoE*-*lacZ* activity to a lesser extent compared to the wild type strain (Fig. 6). This increase was nevertheless still statistically significant (p < 0.01).

### H-NS binds to the *vieSAB*, *tnaA*, *rtxCA*, *rtxBDE* and *hlyA* promoters

**Fig 7:**
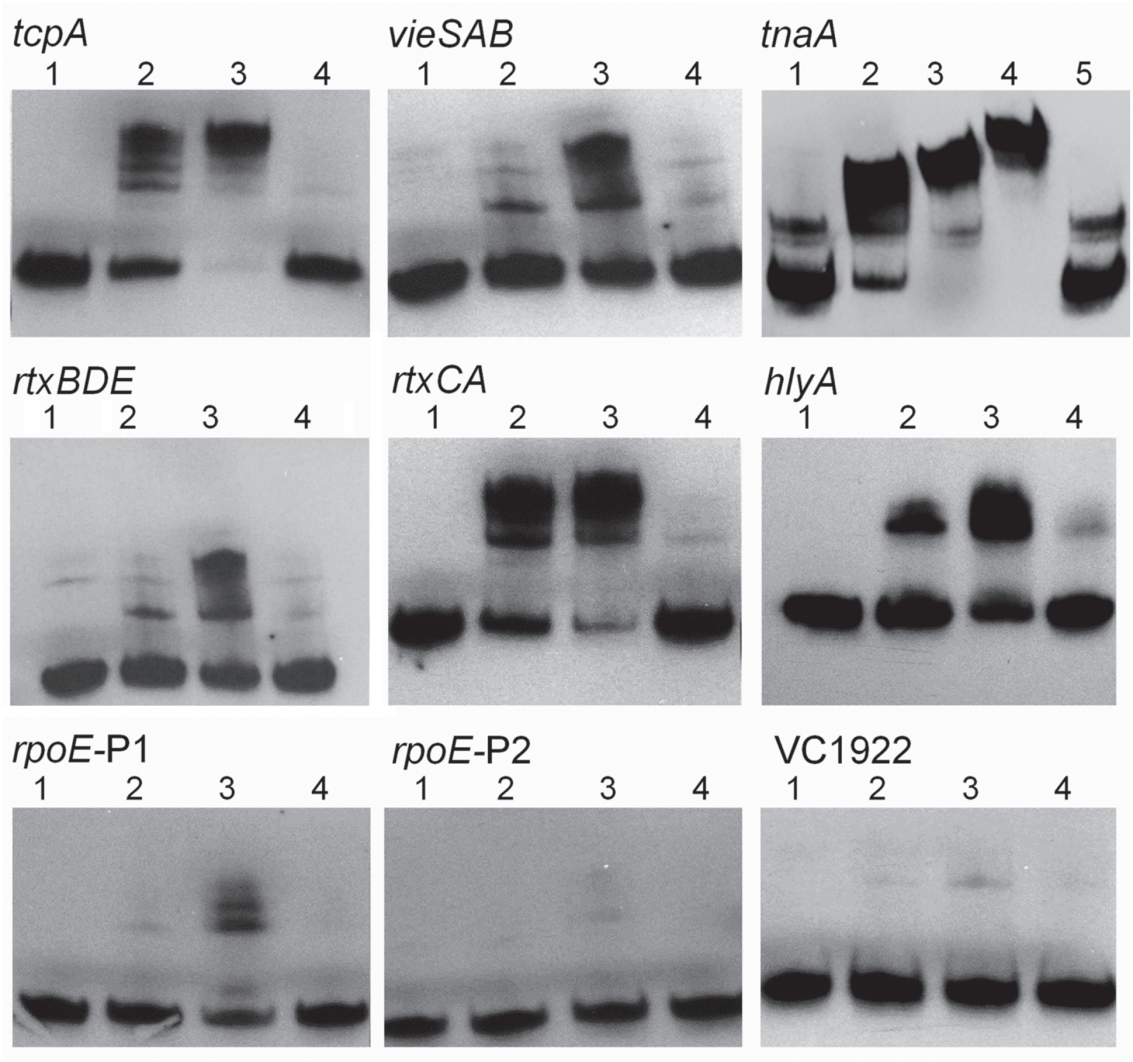
H-NS can bind to the *vieSAB*, *tnaA*, *rtx* and *hlyA* promoters. DIG-labeled DNA fragments containing the *tcpA*, *vieSAB*, *tnaA*, *rtxBDE*, *rtxCA*, *hlyA*, *rpoE-P1*, *rpoE-P2* and VC1922 promoters were incubated with 0 (lane1), 10 (lane 2), 20 (lane 3) and 25 (lane 4 in *tnaA* panel) ng of H-NS protein. The spans of the promoter fragments used relative to the start codon were as follows: *tcpA*, -306 to-83; *vieSAB*, -331 to +21; *tnaA*, -480 to +70; *rtxBDE*, -188 to +39; *rtxCA*, -223 to +24; *hlyA*, -291 to +23; *rpoE* P1, -414 to-230; *rpoE* P2, -180 to-12; VC1922, -113 to +43. Lanes 4 in all panels except *tnaA*, and lane 5 in the *tnaA* panel contained a 100-fold excess of unlabeled competitor promoter DNA. The *tcpA* and VC1922 promoters were used as positive and negative controls, respectively.

**Fig 8:**
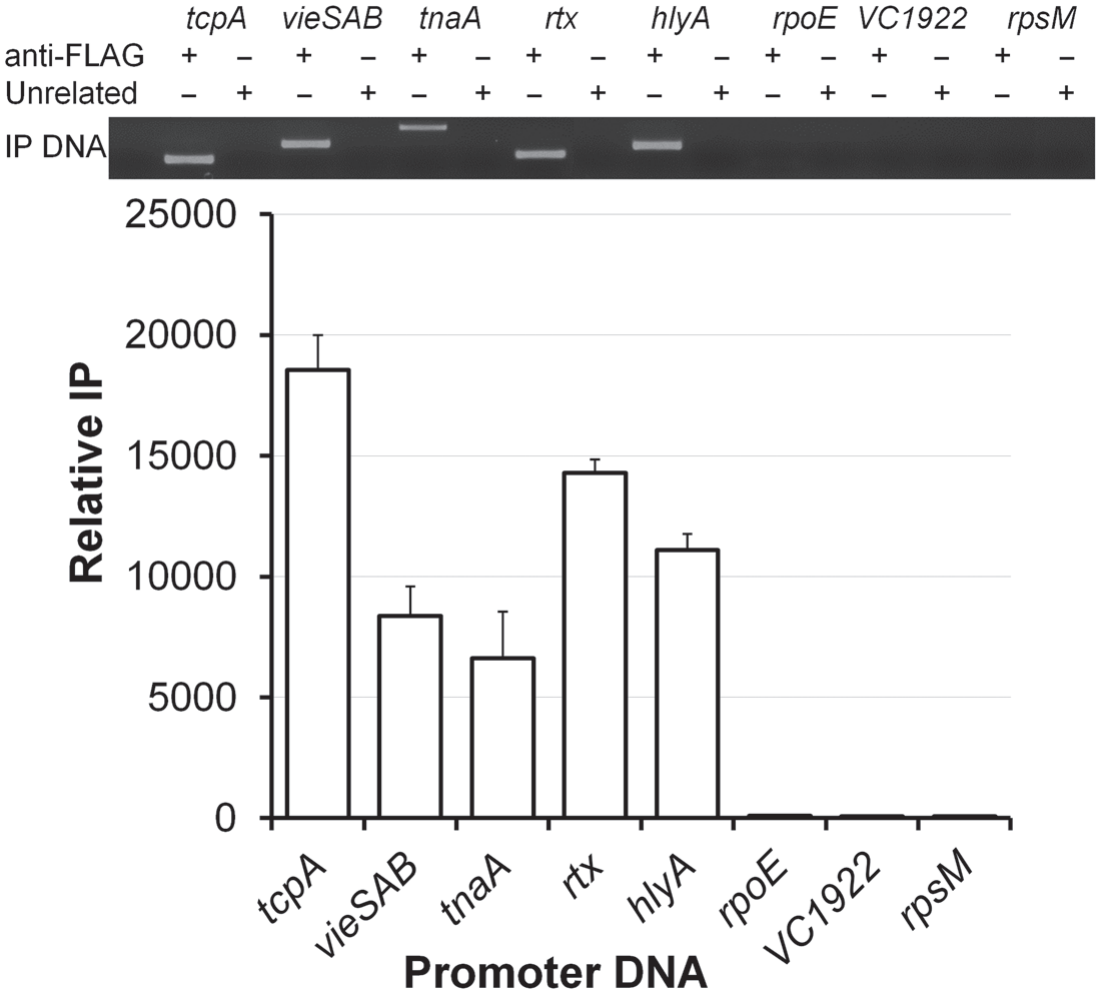
H-NS associates with *vieSAB*, *tnaA*, *rtx* and *hlyA* promoters in the cell. A *V*. *cholerae* strain C7258HNS-FLAG was grown to OD_600_ 0.5. Promoter DNA IP with H-NS was determined by agarose gel electrophoresis (top) and quantitated by qPCR (bottom). Relative IP is the amount of promoter IP with anti-FLAG mAb normalized by the quantity of promoter DNA IP with the unrelated IgG1 isotype control mAb. Positive control, *tcpA*; negatives controls, VC1922, *rpsM*. Each value represents the mean of three experiments. Error bars denote the STDEV.

To determine if H-NS transcriptional repression of the above promoters was direct, we conducted EMSA. As shown in Fig. 7, purified H-NS bound to the *vieSAB*, *tnaA*, *rtxCA*, *rtxBDE and hlyA* promoters but not to the *rpoE* promoter. To determine if H-NS could interact with these promoters in the cell, a condition in which physiological levels of H-NS can co-exist with other transcriptional factors, we conducted ChIP. As shown in Fig. 8, significant H-NS occupancy (similar in magnitude to the *tcpA* promoter used as a positive control) could be demonstrated for all promoters except *rpoE*. In the ChIP assay, the highest occupancies were observed for the *rtx* DNA spanning the intergenic region between the *rtxC* and *rtxB* ORFs and the *hlyA* promoter region.

**Fig 9:**
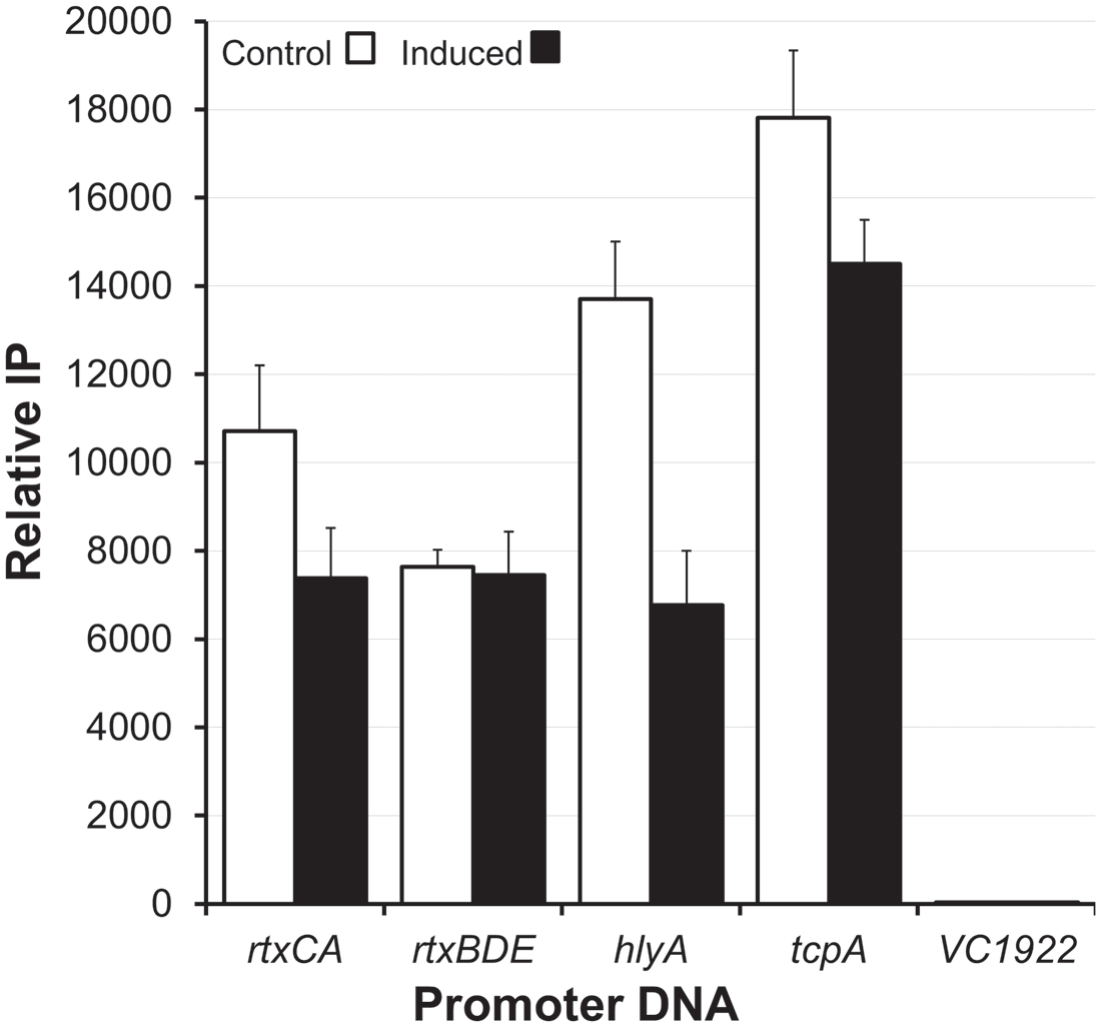
Effect of HlyU on H-NS occupancy at the *rtxCA*, *rtxBDE* and *hlyA* promoters. *V*. *cholerae* strain C7258HNS-FLAG containing pBAD-HlyU-His was grown to OD_600_ 0.5 and the cultures divided in halves. One half was used as a control and the other half treated with 0.02% L-arabinose to induce HlyU expression. Cultures were incubated as described in materials and methods and the amount of promoter DNA IP with H-NS was quantitated by qPCR. Positive control, *tcpA*; negative controls, VC1922. Each value represents the mean of three experiments. Error bars denote the STDEV.

We used a ChIP assay to clarify the interaction between H-NS and HlyU at the *hlyA*, *rtxCA*, and *rtxBDE* promoters. To this end, we overexpressed HlyU from the arabinose promoter in plasmid pBAD-HlyU-His and determined H-NS occupancy at the above promoters. As shown in Fig. 9, overexpression of HlyU had a minor effect on H-NS occupancy at the *rtx* promoters that did not reach statistical significance. Similarly, overexpression of HlyU did not affect H-NS occupancy at the *tcpA*, promoter, which is not regulated by HlyU. In contrast, overexpression of HlyU diminished H-NS occupancy at the *hlyA* promoter (p = 0.011) (Fig. 9). It should be noted that the fragment amplified in this ChIP assay exhibited positive H-NS binding in EMSA. These results suggests that HlyU could act as an H-NS anti-repressor for the *hlyA* gene but not for *rtx* as reported for the *V*. *vulnificus rtxA1* locus [55].

### H-NS positively regulates chemotaxis

**Fig 10:**
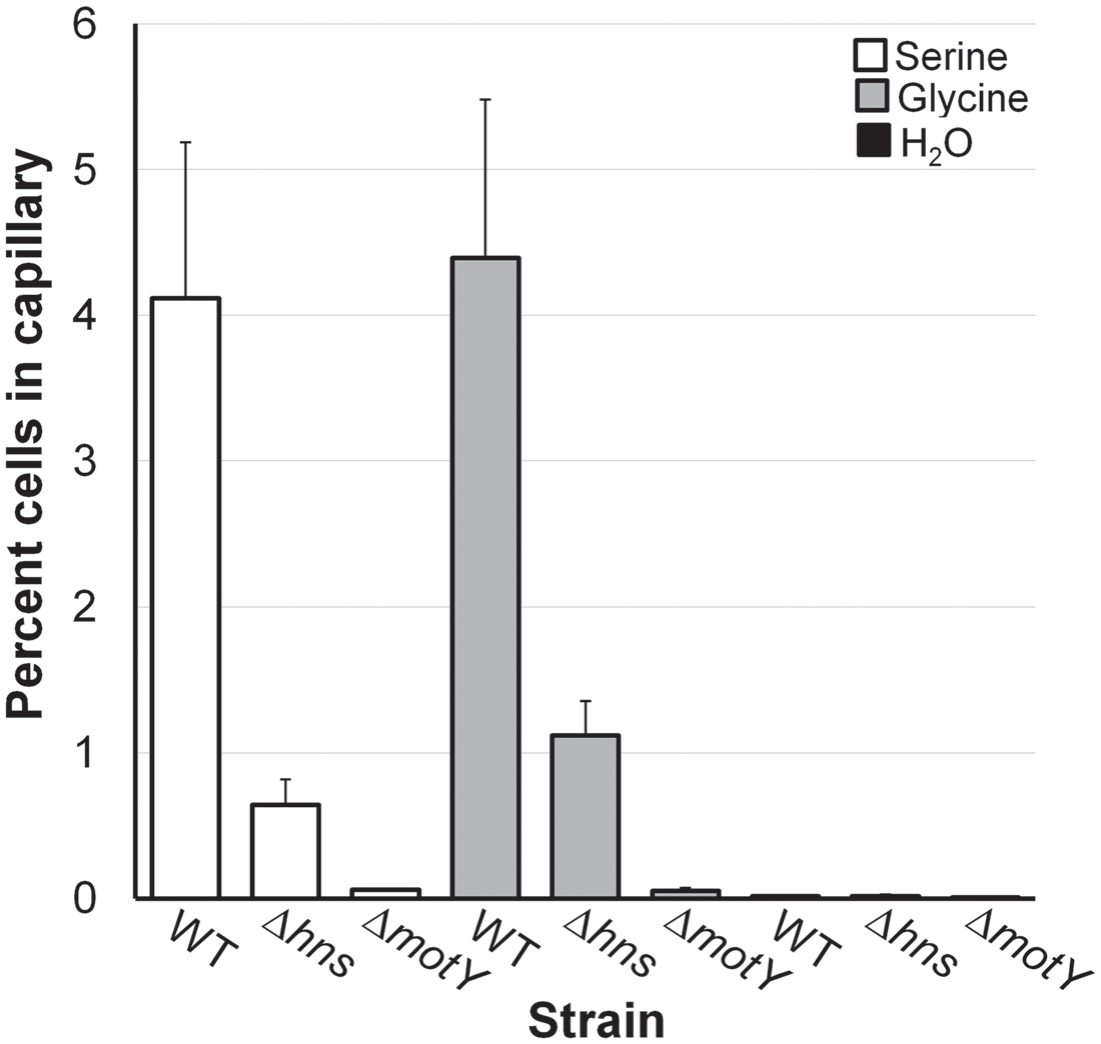
Deletion of *hns* diminishes chemotaxis. A chemotaxis capillary assay was used to measure WT and mutant taxis toward amino acids as described in materials and methods. A non-motile (*motY*) mutant and water were used as negative controls. Each value represents the mean of three experiments. The error bars denote the STDEV.

A remarkable characteristic of the Δ*hns* transcriptome is the lower expression of chemotaxis genes in the early stationary phase (Table 3). This included a broad repertoire of loci annotated as methyl-accepting chemotaxis proteins (MCP), response regulators, histidine kinases, coupling proteins, methyl transferases and methyl esterases [60]. Since the function of most of these genes is ill-defined, we conducted a capillary chemotaxis assay to determine if the Δ*hns* mutant has a chemotaxis phenotype. Since serine and glycine have been confirmed to function as *V*. *cholerae* attractants [49], we determined if the *hns* mutant displayed reduced chemotaxis toward these compounds. As shown in Fig. 10, the Δ*hns* mutant displayed reduced taxis toward these amino acids compared to wild type (p < 0.05).

Diminished chemotaxis has been shown to enhance *V*. *cholerae* infectivity and provide a competitive advantage for colonization of the suckling mouse intestine [61]. We conducted a suckling mouse competitive colonization assay to determine if the diminished chemotaxis exhibited by the *hns* mutant had a positive effect on intestinal colonization. However, despite the *hns* mutant exhibiting diminished chemotaxis, the El Tor biotype mutant showed a 2-fold reduction in infant mouse colonization capacity compared to wild type (CI = 0.45, median of 6 mice). An identical competition experiment in LB medium yielded a CI of 0.48 (n = 4). These results suggest that the diminished intestinal colonization exhibited by the *hns* mutant is a general consequence of its reduced growth rate.

## Discussion and Conclusions

Transcription profiling using RNA-seq showed that loss of H-NS has a significant impact on the transcriptome affecting the expression of 18% of all predicted genes. Here we identify new genes involved in chemotaxis, cell envelope biogenesis, biofilm formation and virulence that are regulated by H-NS. In some cases, the effect of H-NS was found to be growth phase-dependent. H-NS is one of the most abundant proteins in the cell and it is expressed at comparable levels at low cell density, high cell density and early stationary phase [32]. Therefore, the growth phase differences in the number and kind of H-NS responsive genes is not due to differences in H-NS expression. Instead, these differences most likely reflect the expression of other nucleoid-associated proteins that could counteract H-NS repression. For instance, we have shown that induction of IHF in the stationary phase results in lower H-NS occupancy at the *flrA* and *rpoN* promoters [25]. FIS, which has been reported to counteract H-NS repression at several promoters [12, 13], declines sharply at high cell density in *V*. *cholerae* [62]. DPS (DNA protection during starvation), which is expressed in the stationary phase, could indirectly affect H-NS repression due to its effect on DNA topology which changes from negatively supercoiled in log phase cells to more relaxed in stationary phase cells [63]. The activity of CRP known to counteract H-NS repression at a number of promoters [12,13] is regulated by carbon source availability and could increase in stationary phase due to nutrient limitation [64]. Further, RNA polymerase containing σ^S^, expressed in the stationary phase, has been reported to be more resistant to inhibition of transcription initiation by H-NS [65].

The *vieSAB* regulatory system is a regulator of CT biosynthesis, the expression of which differs between *V*. *cholerae* biotypes [3]. This operon is expressed at a higher level in the classical biotype and 20% of the genes that are differentially expressed in the classical versus El Tor biotype are controlled by VieA [3]. In fact, VieA was found to regulate the expression of 10% of the classical biotype genome [3] suggesting that this regulator is a major contributor to the phenotypic differences observed between *V*. *cholerae* biotypes. Here we show that H-NS represses the *vieSAB* operon by binding to its promoter *in vitro* and *in vivo*. Overexpression of the *vieSAB* operon in the El Tor Δ*hns* mutant revealed a new layer of H-NS transcriptional silencing of virulence gene expression. In agreement with previous studies, deletion of *vieA* and *vieSAB* resulted in identical phenotypes suggesting that VieA is the component of this regulatory system that enhances toxin expression [53]. We note, however, that deletion of *vieA* and *vieSAB* diminished *ctxAB* expression only in the *hns* mutant. Overexpression of VieA from a heterologous promoter to bypass H-NS repression had no effect on *ctxAB* expression in wild type *V*. *cholerae* (data not shown). This result could be explained by H-NS transcriptional silencing of the ToxR regulatory cascade at multiple steps [28]. Since *vieA* mutants express diminished *toxT* mRNA [52], our data are consistent with VieA acting at the level of ToxT to enhance *ctxAB* expression when H-NS is absent. Despite *V*. *cholerae* of the El Tor biotype expressing lower levels of *vieSAB* compared to the classical biotype [3], the regions preceding the *vieS* ORF in the two biotypes aligned with ClustalW2 (http://www.ebi.ac.uk/Tools/msa/clustalw2/) are 99% identical. Furthermore, the virtual footprint software (http://www.prodoric.de/vfp) [66] identified three identically spaced H-NS putative binding sites upstream of *vieS* in both biotypes suggesting that H-NS can potentially bind to the *vieSAB* promoter in both biotypes. In spite of the promoter similarity, the same *vieSAB-lacZ* constructs exhibited much higher activities in the classical versus the El Tor biotype. The classical biotype strain used in this study differs from the El Tor strain in that the former does not express the quorum sensing regulator HapR. Here we show that the *vieSAB* operon is also repressed by quorum sensing. We hypothesize that HapR could bind the *vieSAB* promoter to diminish transcription activity together with H-NS in a roughly additive manner. Binding of H-NS and HapR to negatively regulate transcription has been reported for the *vpsT* promoter [32, 67]. A DNA sequence closely matching the HapR binding motif-1 described in [68] is located upstream the *vieSAB* ORF. Alternatively, HapR could act indirectly by inhibiting the expression and/or activity of a positive factor that counteracts H-NS repression.

The El Tor biotype Δ*hns* mutant over expressed additional toxic factors with demonstrated activities in cell culture. This is the case of hemolysin exhibiting pore-forming and vacuolating activities [69] and the RTX toxin causing actin depolymerization [2, 70]. The genes encoding these toxins were expressed at a low level in the wild type strain but were significantly over expressed in the Δ*hns* mutant. DNA binding data, and ChIP showed that repression of *hlyA* and *rtx* genes by H-NS is direct. Repression of *hlyA* by H-NS has also been reported in *V*. *anguillarum* [71]. Interestingly, regulation of *hlyA* expression by iron is lost in the Δ*hns* mutant which, together with the RNA-seq data, suggests that loss of H-NS is accompanied by significant disruption of iron homeostasis. Expressions of *hlyA* in *V*. *cholerae* and *rtx* genes in *V*. *vulnificus* are positively regulated by the transcriptional factor HlyU [54, 55]. We conducted a ChIP assay to examine the effect of HlyU on H-NS occupancy at the *hlyA* and *rtx* promoters. For the *hlyA* gene, the virtual footprint predicts the occurrence of two potential H-NS binding sites separated by 115 bp, one located downstream from the start codon. Such an arrangement suggests the formation of the reported DNA: H-NS: DNA bridge structure trapping the RNA polymerase at promoters [21]. Overexpression of HlyU diminished H-NS occupancy at a *hlyA* promoter fragment spanning the putative H-NS binding site. Since binding of HlyU to the *hlyA* promoter has not been demonstrated [55], we suggest that HlyU diminishes H-NS occupancy indirectly through a hitherto unknown regulator.

Our data are not consistent with HlyU acting as an H-NS antirepressor at the *V*. *cholerae rtx* promoters as proposed for the *V*. *vulnificus rtxA1* promoter [55]. The HlyU proteins of *V*. *vulnificus* and *V*. *cholerae* are closely homologous (93% similarity, 82% identity). However, alignment of the region protected by HlyU at the *V*. *vulnificus rtxA*1 promoter to the DNA located between the *rtxCA* and *rtxBDE* operons in *V*. *cholerae* using the ClustalW2 software did not reveal significant homology. Furthermore, we expressed and purified *V*. *cholerae* HlyU and could not demonstrate binding of this protein to the *rtxCA* and *rtxBDE* promoters by EMSA (data not shown).

Here we show that H-NS binds to the *tnaA* promoter to repress tryptophanase and indole production, a positive regulator of biofilm formation [46]. It has been proposed that indole enhances the expression of *vps* genes required for biofilm matrix polysaccharide biosynthesis [46]. Thus, our studies indicate that H-NS represses biofilm development at two levels: direct repression of *vps* genes [32] and lowering of indole production. Consistent, with a previous transcription profiling of a Δ*crp* mutant [56], we found that the *tnaA* gene is activated by CRP. In *E*. *coli*, expression of *tnaA* is subject to complex regulation that involves CRP transcriptional activation and tryptophan-induced translation anti-termination [72]. The organization of the *tnaA* region in *V*. *cholerae* chromosome II resembles that of *E*. *coli* where the *tnaA* ORF is preceded by a large mRNA leader that includes a sequence encoding the TnaC leader peptide (annotated VCA0161.1 in *V*. *cholerae*). In *E*. *coli*, transcription is initiated upstream of *tnaC* upon activation by the CRP-cAMP complex [72]. Inspection of the *V*. *cholerae tnaA* region with the virtual footprint software showed the presence of a CRP binding site upstream of the leader peptide encoding sequence consistent with the regulation of our *tnaA*-*lacZ* fusion. In contrast, H-NS binding sites predicted by this software were only found downstream of the leader peptide. On this basis, it is unlikely that CRP acts at this promoter by counteracting H-NS repression. This interpretation is consistent with our finding that CRP was still required for maximal *tnaA* expression in the absence of H-NS.

Transcriptional profiling of the Δ*hns* mutant indicated that loss of H-NS results in significant changes in OMP transcription, genes involved in iron binding and transport and oxidative stress. Perturbation of the cell envelope has been reported to result in alterations of iron homeostasis and oxidative stress [57]. Our results are consistent with previous observations that an *hns* mutant expressed elevated catalase and peroxidase encoding genes [27]. Thus, our data suggest that mutational loss of H-NS has a significant impact on the cell envelope. Accordingly, the Δ*hns* mutant overexpressed *rpoE* encoding σ^E^, which is required for virulence in the cholera bacterium [37]. We propose that altered OMP transcription in the Δ*hns* mutant induces an endogenous envelope stress response resulting in σ^E^ release from the inner membrane and activation of its own promoter. In agreement with this interpretation, we did not observe a direct interaction between H-NS and the *rpoE* promoter. Similar to *E*. *coli*, the genes encoding σ^E^, the negative regulators of its activity RseA and RseB, as well as RseC are organized in an operon [58, 59]. Consistent with this organization, genes *rseA*, *rseB* and *rseC* were also overexpressed in the Δ*hns* mutant. However, analogous to *E*. *coli*, it is likely that RseA is proteolytically degraded under conditions of envelope stress [58, 59].

A remarkable feature of the Δ*hns* transcriptome is the down-regulation of numerous genes predicted to affect chemotaxis. This result explains the diminished motility of El Tor biotype *hns* mutants in the swarm agar assay in spite of being flagellated [25, 27]. It has been suggested that H-NS affects gene expression indirectly or by post-transcriptional mechanisms when acting as a positive regulator [12, 13]. Chemotaxis is critical for *V*. *cholerae* to adapt to environmental changes and compete with other bacteria by sensing and responding to chemical gradients. In particular, the MCP family of proteins down-regulated in the *hns* mutant are predicted to sense environmental cues and transduce this information to the flagellar motor through the participation of additional *che* genes, allowing the bacterium to swim toward favorable substrata or away from toxic environments. Down-regulation of these genes in the *hns* mutant could severely hamper its survival capacity in nature. We note that of the several *che* genes down-regulated in the Δ*hns* mutant, only *cheZ* is located in the cluster II reported to be essential for controlling the direction of flagellar rotation [60]. One MCP (VCA1056) down-regulated in the Δ*hns* mutant was reported to be induced upon human infection [49]. Although the function of the remaining MCP and *che* genes down-regulated in the *hns* mutant are not defined, we confirmed that the *hns* mutant exhibits reduced chemotaxis toward glycine and serine.

In the suckling mouse, diminished chemotaxis would be expected to confer a competitive advantage to the *hns* mutant [61]. Nevertheless, the *hns* mutant colonized less compared to wild type, suggesting that loss of other H-NS functions outweighs the potential advantage of being less chemotactic in this model. Loss of H-NS in the El Tor biotype mutant, however, had a less severe effect on intestinal colonization compared to the classical biotype [73].

From the studies described in this article we conclude that:

- H-NS regulates the expression of a significant fraction of the cholera bacterium’s genome in a growth phase-dependent manner. Salient features of the *V*. *cholerae hns* transcriptome are the down-regulation of multiple genes annotated as methyl-accepting chemotaxis proteins resulting in diminished chemotaxis, and the aberrant expression of porins with induction of an endogenous envelope stress response.
- H-NS, in conjunction with quorum sensing, silences the transcription of the VieSAB three-component regulatory system, a major contributor to biotype-specific differences in gene expression.
- H-NS directly repressed the transcription of hemolysin, the RTX toxin and the RTX toxin transport system and indole biosynthesis. Transcriptional silencing of hemolysin (*hlyA*) could be partially counteracted by the transcriptional activator HlyU.
- The identification of new genes silenced by H-NS encoding virulence regulators and known cytotoxic factors that are differentially expressed in *V*. *cholerae* biotypes could facilitate the discovery of additional transcription factors that may contribute to the emergence of new pathogenic variants of the cholera bacterium by anti-silencing.

## Supporting Information

S1 Fig Genes regulated by H-NS by role category. (TIF)

S1 Table Genes differentially expressed in hns mutant in mid-exponential phase. (XLSX)

S2 Table Genes differentially expressed in hns mutant in early stationary phase. (XLSX)

## References

[1] Kaper JB, Morris G Jr, Levine MM. Cholera. Clin Microbiol Rev. 1995;8: 48–86. 7704895 10.1128/cmr.8.1.48 PMC172849

[2] Lin W, Fullner KJ, Clayton R, Sexton JA, Rogers MB, Calia KE, et al Identification of a Vibrio cholerae RTX toxin gene cluster that is tightly linked to the cholera toxin prophage. Proc Natl Acad Sci USA. 1999;96: 1071–1076. 9927695 10.1073/pnas.96.3.1071 PMC15352

[3] Beyhan S, Tischler AD, Camilli A, Yildiz FH. Differences in gene expression between the classical and El Tor biotypes of Vibrio cholerae O1. Infect Immun. 2006;74: 3633–3642. 16714595 10.1128/IAI.01750-05 PMC1479229

[4] Herrington DA, Hall RH, Losonsky GA, Mekalanos JJ, Taylor RK, Levine MM. Toxin, the toxin co-regulated pili and the toxR regulon are essential for Vibrio cholerae pathogenesis in humans. J Exp Med. 1988;168: 1487–1492. 2902187 10.1084/jem.168.4.1487 PMC2189073

[5] Matz C, McDougald D, Moreno AM, Yung PY, Yildiz FH, et al Biofilm formation and phenotypic variation enhance predation-driven persistence of Vibrio cholerae. Proc Natl Acad Sci USA. 2005;102: 16819–16824. 16267135 10.1073/pnas.0505350102 PMC1283802

[6] Jensen MA, Faruque SM, Mekalanos JJ, Levin BR. Modeling the role of bacteriophage in the control of cholera outbreaks. Proc Natl Acad Sci USA. 2006;103: 4652–4657. 16537404 10.1073/pnas.0600166103 PMC1450226

[7] Faruque SM, Naser IB, Islam MJ, Faruque AS, Ghosh AN, Nair GB, et al Seasonal epidemics of cholera inversely correlate with the prevalence of environmental cholera phages. Proc Natl Acad Sci USA. 2005;102: 1702–1707. 15653771 10.1073/pnas.0408992102 PMC547864

[8] Faruque SM, Islam MJ, Ahmad QS, Faruque AS, Sack DA, Nair GB, et al Self-limiting nature of seasonal cholera epidemics: role host-mediated amplification of phage. Proc Natl Acad Sci USA. 2005;102: 6119–6124. 15829587 10.1073/pnas.0502069102 PMC1087956

[9] Faruque SM, Biswas K, Nashir Udden SM, Ahmad QS, Sack DA, Nair GB, et al Mekalanos JJ. Transmissibility of cholera: in vivo formed biofilms and their relationship to infectivity and persistence in the environment. Proc Natl Acad Sci USA. 2006;103: 6350–6355. 16601099 10.1073/pnas.0601277103 PMC1458881

[10] Schoolnik GK, Yildiz FH. The complete genome sequence of Vibrio cholerae: a tale of two chromosomes and two lifestyles. Genome Biol. 2000;1: reviews 1016.1–1016.3. 10.1186/gb-2000-1-3-reviews1016 PMC138858 11178241

[11] Tamayo R, Patimalla B, Camilli A. Growth in a biofilm induces a hyperinfective phenotype in Vibrio cholerae. Infect Immun. 2010;78: 3560–3569. 10.1128/IAI.00048-10 20515927 PMC2916270

[12] Atlung T, Ingmer H. H-NS: a modulator of environmentally regulated gene expression. Mol Microbiol. 1997;24: 7–17. 9140961 10.1046/j.1365-2958.1997.3151679.x

[13] Dorman CJ. H-NS: a universal regulator for a dynamic genome. Nature Rev Microbiol 2004;2: 391–400. 15100692 10.1038/nrmicro883

[14] Cerdan R, Bloch V, Yang Y, Bertin P, Dumas C, Rimsky S, et al Crystal structure of the N-terminal dimerization domain of VicH, the H-NS-like protein of Vibrio cholerae. J Mol Biol. 2003;334: 179–185. 14607110 10.1016/j.jmb.2003.09.051

[15] Nye MB, Taylor RK. Vibrio cholerae H-NS domain structure and function with respect to transcriptional repression of ToxR regulon genes reveals differences among H-NS family members. Mol Microbiol. 2003;50: 427–444. 14617169 10.1046/j.1365-2958.2003.03701.x

[16] Spurio R, Falconi M, Brandi A, Pon CL, Gualerzi CO. The oligomeric structure of the nucleoid protein H-NS is necessary for recognition of intrinsically curved DNA and for DNA binding. EMBO J. 1997;16: 1795–1805. 9130723 10.1093/emboj/16.7.1795 PMC1169782

[17] Dame RT, Wyman C, Goosen N. Structural basis for preferential binding of H-NS to curved DNA. Biochimie 2001;83: 231–234. 11278073 10.1016/s0300-9084(00)01213-x

[18] Lang B, Blot N, Bouffartigues E, Buckle M, Geertz M, Gualerzi CO, et al High-affinity DNA binding sites for H-NS provide a molecular basis for selective silencing within proteobacterial genomes. Nucleic Acids Res. 2007;35: 6330–6337. 17881364 10.1093/nar/gkm712 PMC2094087

[19] Owen-Hughes TA, Pavitt GD, Santos DS, Sidebotham JM, Hulton CSJ, Hinton JCD. et al The chromatin-associated protein H-NS interacts with curved DNA to influence DNA topology and gene expression. Cell. 1992;71: 255–265. 1423593 10.1016/0092-8674(92)90354-f

[20] Ueguchi C, Mizuno T. The Escherichia coli nucleoid protein H-NS functions directly as a transcriptional repressor. EMBO J. 1993;12: 1039–1046. 8458322 10.1002/j.1460-2075.1993.tb05745.x PMC413305

[21] Dorman CJ, Kane KA. Bridging and anti-bridging: a role for bacterial nucleoid-associated proteins in regulating the expression of laterally acquired genes. FEMS Microbiol Rev. 2009;33: 587–592. 10.1111/j.1574-6976.2008.00155.x 19207739

[22] Yu RR, DiRita VJ. Regulation of gene expression in Vibrio cholerae by toxT involves both antirepression and RNA polymerase stimulation. Mol Microbiol. 2002;43: 119–134. 11849541 10.1046/j.1365-2958.2002.02721.x

[23] Stonehouse EA, Kovacikova G, Taylor RK, Skorupski K. Integration host factor positively regulates virulence gene expression in Vibrio cholerae. J Bacteriol. 2008;190: 4736–4748. 10.1128/JB.00089-08 18456804 PMC2446820

[24] Stonehouse EA, Hulbert RR, Nye MB, Skorupski K, Taylor RK. H-NS binding and repression of the ctx promoter in Vibrio cholerae. J Bacteriol. 2011;193: 979–988. 10.1128/JB.01343-09 21169492 PMC3028689

[25] Wang H, Ayala JC, Benitez JA, Silva, AJ. Interaction of the histone-like nucleoid structuring protein and the general stress response regulator RpoS at Vibrio cholerae promoters that regulate motility and hemagglutinin/protease expression. J Bacteriol. 2012;194: 1205–1215. 10.1128/JB.05900-11 22194453 PMC3294804

[26] Tending C, Badaut C, Krin E, Gounon P, Ngo S, Danchin A, et al Isolation and characterization of vicH, encoding a new pleiotropic regulator in Vibrio cholerae. J Bacteriol. 2000;182: 2006–2032. 10.1128/jb.182.7.2026-2032.2000 PMC101921 10715012

[27] Silva AJ, Sultan SZ, Liang W, Benitez JA. Role of the histone-like nucleoid structuring protein (H-NS) in the regulation of RpoS and RpoS-dependent genes in Vibrio cholerae. J Bacteriol. 2008;190: 7335–7345. 10.1128/JB.00360-08 18790865 PMC2576668

[28] Nye MB, Pfau JD, Skorupski K, Taylor RK. Vibrio cholerae H-NS silences virulence gene expression at multiple steps in the ToxR regulatory cascade. J Bacteriol. 2000;182: 4295–4303. 10894740 10.1128/jb.182.15.4295-4303.2000 PMC101945

[29] De Lorenzo V, Eltis L, Kessler B, Timmis KN. Analysis of the Pseudomonas gene products using lacIq/Ptrp-lac plasmids and transposons that confer conditional phenotypes. Gene. 1993;123: 17–24. 8380783 10.1016/0378-1119(93)90533-9

[30] Iwanaga M, Yamamoto K, Higa N, Ichinose Y, Nakasone N, Tanabe M. Culture conditions for stimulating cholera toxin production by Vibrio cholerae O1 El Tor. Microbiol Immunol. 1986;30: 1075–1083. 3543624 10.1111/j.1348-0421.1986.tb03037.x

[31] Donnenberg MS, Kaper JB. Construction of an eae deletion mutant of enteropathogenic Escherichia coli by using a positive selection suicide vector. Infect Immun. 1991;59: 4310–4317. 1937792 10.1128/iai.59.12.4310-4317.1991 PMC259042

[32] Wang H, Ayala JC, Silva AJ, Benitez JA. The histone-like nucleoid structuring protein (H-NS) is a repressor of *Vibrio cholerae* exopolysaccharide biosynthesis (*vps*) genes. Appl Environ Microbiol. 2012;78: 2482–2488. 10.1128/AEM.07629-11 22287003 PMC3302599

[33] Silva AJ, Leitch GJ, Camilli A, Benitez JA. Contribution of hemagglutinin/protease and motility to the pathogenesis of El Tor biotype cholera. Infect Immun. 2006;74: 2072–2079. 16552036 10.1128/IAI.74.4.2072-2079.2006 PMC1418906

[34] Wang H, Ayala JC, Benitez JA, Silva AJ. The LuxR-type regulator VpsT negatively controls the transcription of rpoS encoding the general stress response regulator in Vibrio cholerae biofilms. J Bacteriol. 2014;196: 1020–1030. 10.1128/JB.00993-13 24363348 PMC3957697

[35] Wang H, Zhang L, Silva AJ, Benitez JA. A quinazoline-2,4-diamino analog suppresses Vibrio cholerae flagellar motility by interacting with motor protein PomB and induces envelope stress. Antimicrob Agents Chemother. 2013;57: 3950–3959. 10.1128/AAC.00473-13 23733460 PMC3719709

[36] Boardman BK, Meehan BM, Fullner-Satchell KJ. Growth Phase Regulation of Vibrio cholerae RTX Toxin Export. J Bacteriol. 2007;189: 1827–1835. 17189368 10.1128/JB.01766-06 PMC1855747

[37] Kovacikova G, Skorupski K. The alternative sigma factor sigma (E) plays an important role in intestinal survival and virulence in Vibrio cholerae. Infect Immun. 2002;70: 5355–5362. 12228259 10.1128/IAI.70.10.5355-5362.2002 PMC128310

[38] Silva AJ, Pham K, Benitez JA. Haemagglutinin/protease expression and mucin gel penetration in El Tor biotype Vibrio cholerae. Microbiology. 2003;149: 1883–891. 12855739 10.1099/mic.0.26086-0

[39] Rothmel RD, Shinabarger D, Parsek M, Aldrich T, Chakrabarty AM. Functional analysis of the Pseudomonas putida regulatory protein CatR: transcriptional studies and determination of the CatR DNA binding site by hydroxyl-radical foot printing. J Bacteriol. 1991;173: 4717–4724. 1649820 10.1128/jb.173.15.4717-4724.1991 PMC208149

[40] Stewart V, Yanofsky C. Role of leader peptide synthesis in tryptophanase operon expression in Escherichia coli K-12. J Bacteriol. 1986;167: 383–386. 3522554 10.1128/jb.167.1.383-386.1986 PMC212888

[41] Giannoukos G, Ciulla DM, Huang K, Haas BJ, Izard J, Levin JZ, et al Efficient and robust RNA-seq process for cultured bacteria and complex community transcriptomes. Genome Biol. 2012;13: R23 10.1186/gb-2012-13-3-r23 22455878 PMC3439974

[42] Trapnell C, Pachter L, Salzberg SL. TopHat: discovering splice junctions with RNA-seq. Bioinformatics. 2009;25: 1105–1111. 10.1093/bioinformatics/btp120 19289445 PMC2672628

[43] Trapnell C, Roberts A, Goff L, Pertea G, Kim D, Kelley DR, et al Differential gene and transcript expression analysis of RNA-seq experiments with TopHat and Cufflinks. Nature Protocols. 2012; 7: 562–578. 10.1038/nprot.2012.016 22383036 PMC3334321

[44] Langmead B, Trapnell C, Pop M, Salzberg SL. Ultrafast and memory-efficient alignment of short DNA sequences to the human genome. Genome Biol. 2009;10: R25 10.1186/gb-2009-10-3-r25 19261174 PMC2690996

[45] Guzman LM, Belin D, Carson MJ, Beckwith J. Tight regulation, modulation, and high-level expression by vectors containing the arabinose pBAD promoter. J Bacteriol. 1995;177: 4121–4130. 7608087 10.1128/jb.177.14.4121-4130.1995 PMC177145

[46] Mueller RS, Beyhan S, Saini SG, Yildiz FH, Bartlett DH. Indole acts as an extracellular cue regulating gene expression in *Vibrio cholerae* . J Bacteriol. 2009;191: 3504–3516. 10.1128/JB.01240-08 19329638 PMC2681914

[47] Stroebner JA, Payne SM. Iron-regulated hemolysin production and utilization of heme and hemoglobin by Vibrio cholerae. Infect Immun. 1998;56: 2891–2895. 10.1128/iai.56.11.2891-2895.1988 PMC259667 2971620

[48] Miller JH. Experiments in molecular genetics. Cold Spring Harbor: Cold Spring Harbor Laboratory Press; 1972.

[49] Nishiyama S, Suzuki D, Itoh Y, Suzuki K, Tajima H, Hyakutake A, et al Mlp24 (McpX) of Vibrio cholerae implicated in pathogenicity functions as a chemoreceptor for multiple amino acids. Infect Immun. 2012;80: 3170–3178. 10.1128/IAI.00039-12 22753378 PMC3418727

[50] Kovacikova G, Skorupski K. Overlapping binding sites for the virulence gene regulators AphA, AphB and cAMP-CRP at the Vibrio cholerae tcpPH promoter. Mol Microbiol. 2001;41: 393–407. 11489126 10.1046/j.1365-2958.2001.02518.x

[51] Lee SH, Angelichio MJ, Mekalanos JJ, Camilli A. Nucleotide sequence and spatiotemporal expression of the Vibrio cholerae vieSAB genes during infection. Infect Immun. 1998;180: 2298–2305. 9573178 10.1128/jb.180.9.2298-2305.1998 PMC107168

[52] Tischler AD, Lee SH, Camilli A. The Vibrio cholerae vieSAB locus encodes a pathway contributing to cholera toxin production. J Bacteriol. 2002;184: 4104–4113. 12107127 10.1128/JB.184.15.4104-4113.2002 PMC135224

[53] Tischler AD, Camilli A. Cyclic diguanylate regulates Vibrio cholerae virulence gene expression. Infect Immun. 2005;73: 5873–5882. 16113306 10.1128/IAI.73.9.5873-5882.2005 PMC1231145

[54] Williams SG, Manning PA. Transcription of the Vibrio cholerae haemolysin gene, hlyA, and cloning of a positive regulator locus, hlyU. Mol Microbiol. 1991;5: 2031–2038. 1766378 10.1111/j.1365-2958.1991.tb00825.x

[55] Liu M, Crosa JH. The regulator HlyU, the repeat-in-toxin gene rtxA1, and their roles in the pathogenesis of Vibrio vulnificus infections. MicrobiologyOpen. 2012;4: 502–513. 10.1002/mbo3.48 23233275 PMC3535394

[56] Liang W, Pascual-Montano A, Silva AJ, Benitez JA. The cyclic AMP receptor protein modulates quorum sensing, motility and multiple genes that affect intestinal colonization in Vibrio cholerae. Microbiology. 2007;153: 2964–2975. 17768239 10.1099/mic.0.2007/006668-0

[57] Sikora AE, Beyhan S, Bagdasarian M, Yildiz F, Sandkvist M. Cell Envelope Perturbation Induces Oxidative Stress and Changes in Iron Homeostasis in Vibrio cholerae. J Bacteriol. 2009;191: 5398–5408. 10.1128/JB.00092-09 19542276 PMC2725621

[58] Raivio TL, Silhavy TJ. Periplasmic stress and ECF Sigma factors. Ann Rev Microbiol. 2001;55: 591–624. 11544368 10.1146/annurev.micro.55.1.591

[59] Raivio TL. Envelope stress responses and Gram-negative bacterial pathogenesis. Mol Microbiol. 2005;56: 1119–1128. 15882407 10.1111/j.1365-2958.2005.04625.x

[60] Boin MA, Austin MJ, Hase CC. Chemotaxis in Vibrio cholerae. FEMS Microbiol Lett. 2004;239: 1–8. 15451094 10.1016/j.femsle.2004.08.039

[61] Butler SM, Camilli A. Both chemotaxis and net motility greatly influence the infectivity of Vibrio cholerae. Proc Natl Acad Sci USA. 2004;101: 5018–5023. 15037750 10.1073/pnas.0308052101 PMC387366

[62] Lenz DH, Bassler BL. The small nucleoid protein Fis is involved in Vibrio cholerae quorum sensing Mol Microbiol. 2007;63: 859–871. 17181781 10.1111/j.1365-2958.2006.05545.x

[63] Dorman CJ. Genome architecture and global gene regulation in bacteria: making progress towards a unified model. Nature Rev Microbiol. 2013;11: 349–355. 10.1038/nrmicro3007 23549066

[64] Stulke J, Hillen W. Carbon catabolite repression in bacteria. Curr Opin Microbiol. 1999;2: 195–201. 10322165 10.1016/S1369-5274(99)80034-4

[65] Shin M, Song M, Rhee JH, Hong Y, Kim YJ, Seok YJ, et al DNA looping-mediated repression by histone-like protein H-NS: specific requirement of σ70 as a cofactor for looping. Genes Dev. 2005;19: 2388–2398. 16204188 10.1101/gad.1316305 PMC1240047

[66] Münch R, Hiller K, Grote A, Scheer M, Klein J, Schobert M, et al Virtual Footprint and PRODORIC: an integrative framework for regulon prediction in prokaryotes. Bioinformatics. 2005; 21: 4187–4189. 16109747 10.1093/bioinformatics/bti635

[67] Srivastava D, Harris RC, Waters CM. Integration of cyclic di-GMP and quorum sensing in the control of *vpsT* and *aphA* in *Vibrio cholerae* . J Bacteriol. 2011;193: 6331–5341. 10.1128/JB.05167-11 21926235 PMC3209240

[68] Tsou AM, Cai T, Liu Z, Zhu J, Kulkarni RV. Regulatory targets of quorum sensing in *Vibrio cholerae*: evidence for two distinct HapR-binding motifs. Nucl Acids Res. 2009;37: 2747–2756. 10.1093/nar/gkp121 19276207 PMC2677876

[69] Figueroa-Arredondo P, Heuser JE, Akopyants NS, Hiroshi Morisaki J, Giono-Cerezo S, Enriquez-Rincon J et al Cell vacuolation caused by Vibrio cholerae hemolysin. Infect Immun. 2001;69: 1613–1624. 11179335 10.1128/IAI.69.3.1613-1624.2001 PMC98064

[70] Cordero CL, Kudryashov DS, Reisler E, Fullner Satchell KJ. The actin cross-linking domain of the Vibrio cholerae RTX toxin directly catalyzes the covalent cross-linking of actin. J Biol Chem. 2006;281: 32366–32374. 16954226 10.1074/jbc.M605275200 PMC2255562

[71] Mou X, Spinard EJ, Driscoll MV, Zhao W, Nelson DR. H-NS is a negative regulator of the two hemolysin/cytotoxin gene clusters in Vibrio anguillarum. Infect Immun. 2013;81: 3566–3576. 10.1128/IAI.00506-13 23836825 PMC3811754

[72] Yanofsky C. RNA-based regulation of genes of tryptophan synthesis and degradation in bacteria. RNA. 2007;13: 1141–1154. 17601995 10.1261/rna.620507 PMC1924887

[73] Ghosh A, Paul K, Chowdhury R. Role of the histone-like nucleoid structuring protein in colonization, motility, and bile-dependent repression of virulence gene expression in Vibrio cholerae. Infect Immun. 2006;74: 3060–3064. 16622251 10.1128/IAI.74.5.3060-3064.2006 PMC1459692

