# Supplementary figures and images for: RNA-Seq Analysis Identifies New Genes Regulated by the Histone-Like Nucleoid Structuring Protein (H-NS) Affecting Vibrio cholerae Virulence, Stress Response and Chemotaxis
Source: PLoS One. 2015 Feb 13;10(2):e0118295. doi: 10.1371/journal.pone.0118295 (PMC4332508; doi:10.1371/journal.pone.0118295)

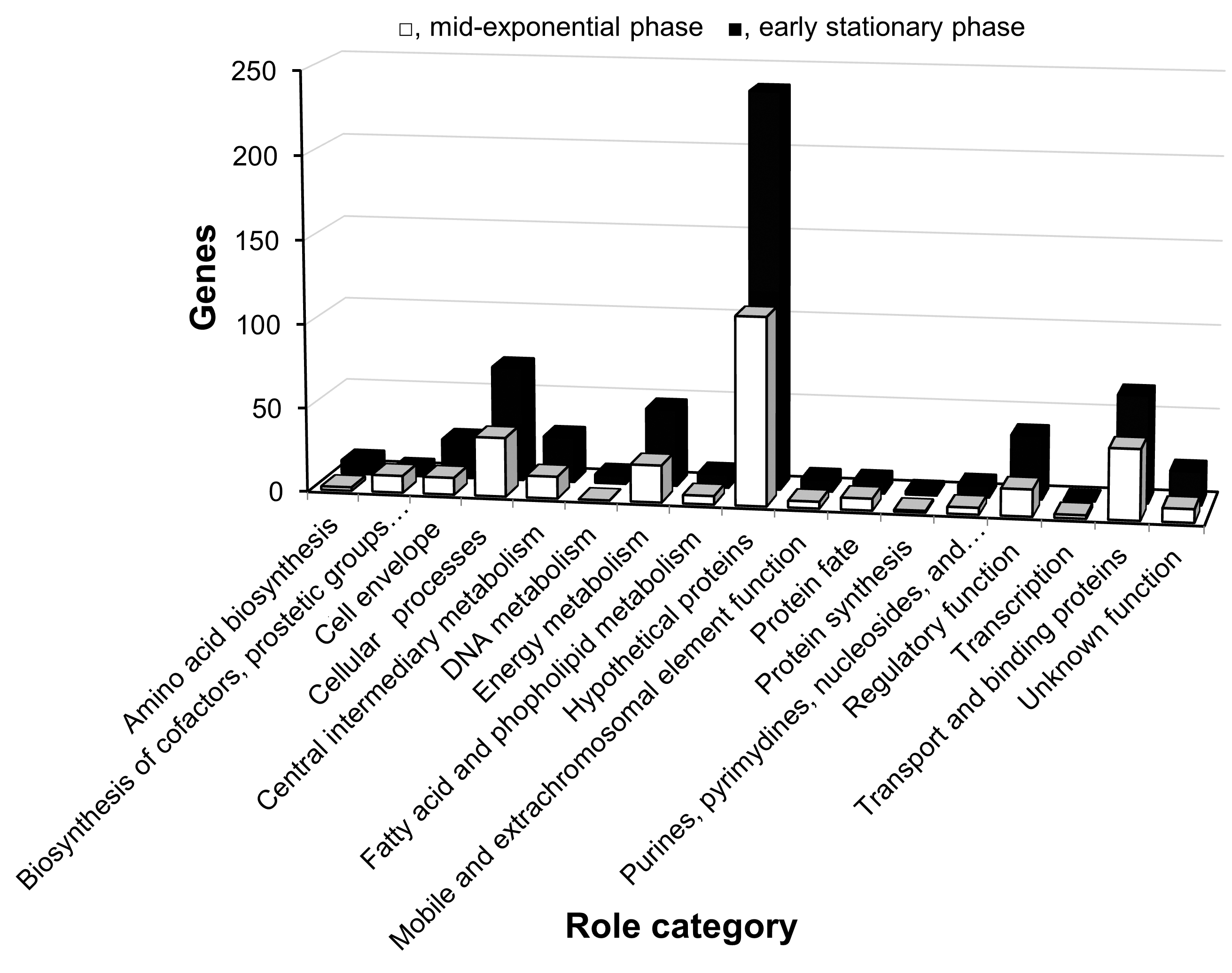

Supplement: S1 Fig — (TIF) [file pone.0118295.s001.tif]
